# Arbuscular Mycorrhiza-Mediated Regulation of Polyamines and Aquaporins During Abiotic Stress: Deep Insights on the Recondite Players

**DOI:** 10.3389/fpls.2021.642101

**Published:** 2021-06-17

**Authors:** Karuna Sharma, Samta Gupta, Sarda Devi Thokchom, Pooja Jangir, Rupam Kapoor

**Affiliations:** Department of Botany, University of Delhi, New Delhi, India

**Keywords:** abiotic stress, arbuscular mycorrhizal fungi, aquaporins, polyamines, salt stress, drought stress, heavy metal toxicity

## Abstract

Environmental stresses of (a)biotic origin induce the production of multitudinous compounds (metabolites and proteins) as protective defense mechanisms in plants. On account of the regulation of some of these compounds, arbuscular mycorrhizal fungi (AMF) reinforce the inherent tolerance of plants toward the stress of different origins and kind. This article reviews two specific fundamental mechanisms that are categorically associated with mycorrhiza in alleviating major abiotic stresses, salt, drought, and heavy metal (HM) toxicity. It puts emphasis on aquaporins (AQPs), the conduits of water and stress signals; and polyamines (PAs), the primordial stress molecules, which are regulated by AMF to assure water, nutrient, ion, and redox homeostasis. Under stressful conditions, AMF-mediated host AQP responses register distinct patterns: an upregulation to encourage water and nutrient uptake; a downregulation to restrict water loss and HM uptake; or no alterations. The patterns thereof are apparently an integrative outcome of the duration, intensity, and type of stress, AMF species, the interaction of fungal AQPs with that of plants, and the host type. However, the cellular and molecular bases of mycorrhizal influence on host AQPs are largely unexplored. The roles of PAs in augmenting the antioxidant defense system and improving the tolerance against oxidative stress are well-evident. However, the precise mechanism by which mycorrhiza accords stress tolerance by influencing the PA metabolism *per se* is abstruse and broadly variable under different stresses and plant species. This review comprehensively analyzes the current state-of-art of the involvement of AMF in “PA and AQP modulation” under abiotic stress and identifies the lesser-explored landscapes, gaps in understanding, and the accompanying challenges. Finally, this review outlines the prospects of AMF in realizing sustainable agriculture and provides insights into potential thrust areas of research on AMF and abiotic stress.

## Introduction

Environmental factors like light, temperature, water, and soil status are important abiotic components that regulate the plant life cycle, and any abnormality in these factors predisposes the plants to stress, a state of altered physiology (Sade et al., 2013). Abiotic stresses, such as salinity, drought, and heavy metals (HMs), result in a 70% reduction in global crop yield worsening the dwindling equilibrium between crop production and exponential population growth (Singh et al., 2016). In addition to this, abiotic stresses, particularly salinity and HMs, depress the nutrient availability in soil and lead to nutritional disorders in plants (Juniper and Abbott, 1993; Evelin et al., 2012; Gusman et al., 2013; Gupta et al., 2021). Soil salinity and HM contamination have been demonstrated to limit the accumulation of minerals, such as Ca, Fe, Mg, and Zn in edible parts of crop plants (Gusman et al., 2013; Chakraborty et al., 2016; Alam et al., 2019; Liu et al., 2020), consequently reducing the nutritive value of the crops. Since these minerals are essential for the human diet, any decrease in their concentrations will have a major impact on nutritional security of food and can give rise to hidden hunger, which is a grave issue especially in a scenario where the global burden of crop production and micronutrient deficiency remains alarming (FAO, 2017).

High soil salinity (>4 dS/m; Juniper and Abbott, 1993) stems from rapid uptake and translocation of Na^+^ and Cl^−^ ions by the plants at the expense of important nutrients such as K, P, and Ca and sequential translocation of these ions into the shoot tissues, thereby increasing the toxic load. Drought stress results from a moderate water loss, which, when gets extensive, results in cellular desiccation (Jaleel et al., 2009) that can cause potential loss of enzyme activity, gross disturbance of plant metabolism, and handicapped cell structure and function. Moreover, essential (Cu, Mn, Zn, Fe, Mo, Co, and Ni) and non-essential (As, Pb, Sn, Cd, Hg, Al, and Cr) HMs also generate toxic/lethal effects on plant growth and metabolism by the inhibition of nutrient assimilation and biomass accumulation, degradation of chlorophyll, passivation of enzymes, disturbed water balance, and senescence. These stresses exacerbate the risk to food security of the world with a teeming population that is projected to mushroom to 11.2 billion by the end of the century (Roser et al., 2013).

Most of the mitigation strategies that are employed to cope with adverse impacts of abiotic stress are either long term, such as breeding stress-tolerant varieties, or are inaccessible or costly to farmers (Bharti et al., 2017). We endeavor to usher sustainable crop production in a way that does not cause economical brunt on farmers. However, noteworthy is the fact that, despite considerable research into the effects of salt and drought on crops, the release of abiotic-tolerant/resistant cultivars has not been commensurate. The reason is most likely due to the complex nature of the effect of stressors on crop plant; as in a multigenic trait, like tolerance to stress, the hierarchy of different aspects of tolerance may differ among and within species (Flowers and Yeo, 1995; Flowers et al., 1997). Abiotic tolerance is way too complex to be easily amenable to refinement through the selection of a trait *per se*. On that account, arbuscular mycorrhizal fungi (AMF) emerge to the occasion as a group of potential stress mitigators in plants owing to their tolerance to extremities, diversity in genetic makeup, ubiquity, and promiscuous interaction with plants and soil (Bharti et al., 2017). In view of that, the deployment of AMF to alleviate abiotic stress in plants is a better, an economical, and an effective agronomic option.

Intrinsic protective mechanisms aside, plants can counteract environmental stress by associating with AMF that form a promiscuous mutualistic relationship with the majority of terrestrial plants (>100,000 species) (Wang and Qiu, 2006). AMF orchestrate various biochemical and physiological pathways that coordinate to impart tolerance to the host plant. They maintain nutrients, ions, and redox homeostasis; enhance water acquisition by serving as extended plant roots, ensure photosynthetic efficiency and osmoregulation, and reinforce antioxidant metabolism (Evelin et al., 2019). Much of these protective roles conferred by mycorrhizal associations during stress can be ascribed to multiple factors, of which AMF-mediated modulation of polyamines (PAs) and aquaporins (AQPs), the recondite players in abiotic stress mitigation, are discussed at length in this review.

Due to their positively charged characteristics, PAs interact with anionic functional groups of membranes and proteins (Slocum et al., 1984). They influence the stability and permeability of cell membranes by the formation of electrostatic bonds with phospholipid head groups (Besford et al., 1993). They have also been reported to prevent loss of chlorophyll from thylakoid membranes by stabilizing the photosystem complexes (Popovic et al., 1979). PAs enhance osmolyte and antioxidant production and interact with other important metabolic routes that are of the essence to stress tolerance in plants. As highlighted in the review, there exists coordination between AMF and the PA metabolism (Sannazzaro et al., 2007; Cicatelli et al., 2010; Wu et al., 2012a; Abdel-Fattah et al., 2013; Talaat and Shawky, 2013; Zhang et al., 2020). However, the understanding of an exact mechanism underlying AMF-mediated modulation of PA pool in relation to salt/drought/HM tolerance is an uncharted area; ergo, merits more research for deeper insights and practical implications. AQPs are intrinsic membrane proteins that facilitate transcellular passive conduction of water and neutral molecules, a significant role that is implicated in the alleviation of water stress. Studies demonstrating the differential regulation of AQP genes in AMF-inoculated plants under water-deficit conditions (salinity and drought) consolidate the role of AMF in helping plants combat water scarcity, but the mechanisms underlying these responses at cellular and molecular level still remain elusive. However, the role of AQPs in combating metal stress in plants is limited to few records and demands extensive exploration. Information on the direct relationship among the two biomolecules (PA and AQP) and AMF *per se* is sketchy. However, it can be speculated that AMF-mediated PA homeostasis could be involved in regulating AQP protein abundance, thereby contributing to the maintenance of plant–water relations; although no concrete evidence exists to reinforce this postulation. Future research should venture into such investigations to expand the dimensions and depth of our understanding on this subject.

Along this review, we explore and analyze the contribution of various studies on the role of AMF in the modulation of the PA metabolism and AQP gene expression as significant mechanisms to impart tolerance to abiotic stresses in plants. As devising strategies that are oriented to improve stress recovery and stress resistance in plants become an enormous challenge, this review will outline the prospects of AMF in realizing sustainable agriculture.

## POLYAMINE Metabolism and Their Cross Talk With Other Metabolic Routes Involved in Stress Tolerance

Polyamines are ubiquitous biostimulants, with low-molecular-weight, that participate in an array of plant growth, developmental and defense processes under stressful conditions (Chen et al., 2019). Triamine spermidine (Spd), tetramine spermine (Spm), and their precursor, diamine putrescine (Put) are the principal free PAs found in plants (Galston and Sawhney, 1990).

**Figure 1:**
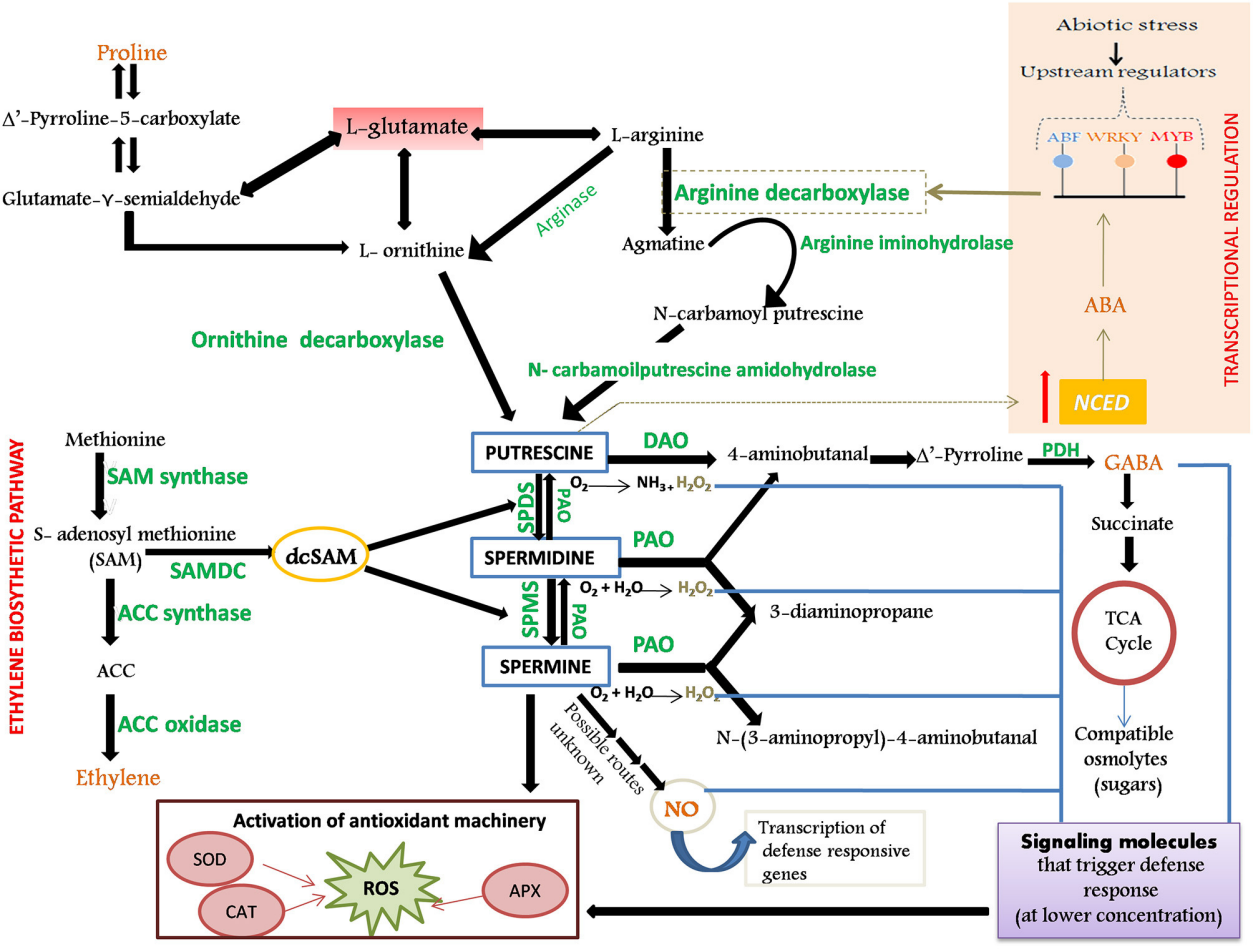
Schematic representation of biosynthesis and catabolism of polyamines (PAs), their role in the activation of defense response in plants and cross-talk with stress-defensive metabolites. The enzymes involved in the metabolic processes are shown in green color. Putrescine (Put), the central molecule of PA biosynthesis, can be synthesized either by arginine (by ADC) or by ornithine (by ODC) derived pathway. Synthesis of diamine spermidine (Spd) and tetramine spermine (Spm) is brought about by the subsequent addition of aminopropyl groups (furnished from the decarboxylated penultimate precursor of ethylene, dcSAM) to the Put skeleton, and is catalyzed by SPDS and SPMS, respectively. Degradation of Put is catalyzed by DAO and that of Spd and Spm by PAO. PAs, along with their catabolic by-products (GABA, H_2_O_2_, and NO), serve as stress messengers and participate in the reinforcement of antioxidant machinery. Production of GABA from PA further boosts the production of compatible osmolytes (such as sugars) under stress. Put and Pro are linked by the common precursor glutamate and share precursor–product relationship regulated by DAO and GABA. Through possibly unknown mechanisms, both PA and NO are found to increase production of each other during stress. Spd possibly intensifies NOS activity that boosts NO production. Put and abscisic acid (ABA) are invested in a positive feedback circuit; Put activates *NCED* gene that triggers ABA accumulation, which further activates TFs that in turn regulate the *ADC* gene. ODC, Ornithine Decarboxylase; ADC, Arginine Decarboxylase; DAO, Diamine Oxidase; PAO, Polyamine Oxidase; PDH, Pyrroline Dehydrogenase; SPMS, Spermine Synthase; SPDS, Spermidine Synthase; ACC, 1-aminocyclopropane-1-carboxylic acid; NO, Nitric Oxide; GABA, Gamma-Aminobutyric Acid; H_2_O_2_, Hydrogen Peroxide; dcSAM, decarboxylated S-Adenosyl Methionine; Pro, Proline; NOS, Nitric Oxide Synthase; *NCED, 9-Cis-Epoxycarotenoid Dioxygenase*.

*PA metabolism*: Putrescine occupies a central position in the common PA biosynthetic pathway whose biosynthesis transpires *via* two parallel routes: (1) arginine-derived pathway involving arginine decarboxylase (ADC) and (2) ornithine-derived pathway involving ornithine decarboxylase (ODC) (Hanfrey et al., 2001; Kakkar and Sawhney, 2002). The aminopropyl residues that are successively incorporated into the Put skeleton to yield Spd and Spm are furnished from decarboxylated S-adenosyl methionine (dcSAM). The addition of the aminopropyl moieties to the Put precursor is effectuated by the enzymes Spd synthase (SPDS) and Spm synthase (SPMS), respectively. The catabolism of PA is impelled by the action of two major classes of amine oxidases: Cu-containing diamine oxidase (DAO) and flavoprotein-containing PA oxidase (PAO). Stringent regulation of cellular PA titers is imperative for evoking responses to environmental cues (such as stress) through an intricate crosstalk with other metabolic pathways that are involved in the synthesis of stress signals and metabolites [ethylene, proline, gamma-aminobutyric acid (GABA), nitric oxide (NO), and abscisic acid (ABA)] involved in amplifying the defense response of plants against encountered stress (Figure 1).

*Cross talk of PA metabolism with stress-defensive metabolites*: Ethylene and PAs are interlinked by a common precursor, S-adenosyl methionine (SAM). The deviation of metabolic flux from ethylene synthesis toward PA synthesis confers tolerance as ethylene temporarily and reversibly hinders cell cycle and speeds up senescence in plants that are under the grip of environmental stress (Even-Chen et al., 1982; Dubois et al., 2018). The synthesis of proline is also in strong correlation with the PA metabolic route by virtue of the common precursor, glutamate (Sannazzaro et al., 2007; Mohapatra et al., 2009). A significant contribution of Put degradation by DAO to proline accumulation has also been reported. The ensuing accumulation of proline by the PA oxidation mediates osmoprotection and serves as a nitrogen reserve that can be exploited by the plant during recovery. Stress-triggered changes in the level of PAs translate into the alteration in cellular content of a non-protein amino acid, GABA, which further boosts the antioxidant capacity and synthesis of protective osmolytes, proline and soluble sugars like trehalose, that are essential for cellular osmotic adjustments (Wang et al., 2017; Priya et al., 2019). PAs also induce the production of a signaling molecule, NO (Tun et al., 2006), that may bridge the PA-mediated stress response with other mediators of stress. NO also, in turn, increases the PA titers under stress (Tailor et al., 2019). PAs, in alliance with their catabolic products, H_2_O_2_ and NO, formed *via* different metabolic routes, appear to act cooperatively to fine-tune ABA responses in stomatal closure during physiological drought conditions (Yamasaki and Cohen, 2006). The presence of ABA-responsive elements, ABRE (namely, ABRE-related motifs) in the promoter sequence of PA biosynthetic genes has reinforced the notion that the participation of PAs in stress tolerance is ABA-dependent. PAs, precisely Put, and ABA are invested in a positive feedback circuit within which they mutually foster production of each other to ameliorate acclimation of plant to abiotic stress (Alcázar et al., 2010). Put activates the *NCED* gene that prompts the ABA accumulation *via* zeaxanthin. This further activates the ABRE elements that stimulate AREB/ABF transcription factors (TFs). These TFs, in turn, regulate the *ADC* gene and direct the synthesis of principal PA, Put (Espasandin et al., 2014) (Figure 1). Additionally, ABA can encourage methionine to produce a generous amount of higher PAs *via* dcSAM (Li et al., 2020).

*Interaction among PAs, phytohormones, and other metabolites*: There exists a highly conserved pathway that is expressed in plants subjected to stress, integrating different phytohormones that regulate plant processes *via* changes in glutamate metabolism (Podlešáková et al., 2018); and therefore, in the PA metabolism, regulating the reactive oxygen species (ROS) responses and NO production, which successively also regulate phytohormone signaling in stressed plants (Freschi, 2013). In general, PAs and plant hormone biosynthesis and signaling are closely related (reviewed by Anwar et al., 2015). Their extensive scrutiny of published data revealed that generally Put is positively correlated with the expression of genes regulating ABA biosynthesis (Alcazar et al., 2005; Singh et al., 2018) but have an inverse effect on hormones, such as ethylene, jasmonic acid, and gibberellic acid (GA) (Alcazar et al., 2005; Cuevas et al., 2008) while the action of Spd is totally antipodal to that (Radhakrishnan and Lee, 2013a; Li Z. et al., 2016). Conversely, Spm promotes the expression of genes involved in ethylene and jasmonate biosynthesis (Ozawa et al., 2009; Gonzalez et al., 2011) while downregulating those for gibberellins (Gonzalez et al., 2011) and ABA biosynthesis (Radhakrishnan and Lee, 2013b). Spd is found to positively regulate salicylic acid (SA) signaling (Lazzarato et al., 2009), and those of cytokinins and auxins are linked to Spm action (Anwar et al., 2015; Sharma et al., 2019). Overall, the nature of these cross-talks between PAs and phytohormones varies with the set of PAs and the hormone involved, and also with the developmental stage of the plant and abiotic conditions to which it is subjected. Mycorrhizal plants perceive the stress cues that trigger the modification in the endogenous levels of phytohormones and/or PA (phyto-regulators). This influences the expression of genes involved in primary metabolism and those relating to amino acid metabolism (proline, GABA, and glycine betaine), carbohydrate metabolism (trehalose and sucrose), and antioxidant metabolism [ascorbate–glutathione (AsA-GSH) cycle and enzymatic antioxidant defense]. AMF-protected plants exhibit reinforced PA production (Sannazzaro et al., 2007; Evelin et al., 2013), upregulated osmolyte production (Evelin et al., 2013; Garg and Saroy, 2020), and regulated synthesis of phytohormones (Shaul-Keinan et al., 2002; Khalloufi et al., 2017; Ren et al., 2018), all of which might potentially interconnect at various levels and ameliorate the tolerance mechanism for cumulative stress response (Hashem et al., 2018). Different phytohormones influence overlapping processes such that the result of phytohormone action relies on a certain hormone combination rather than on their discrete actions (Iqbal et al., 2014).

## AMF-Mediated Modulation of POLYAMINES and Their Responses Under Stress

**Figure 2:**
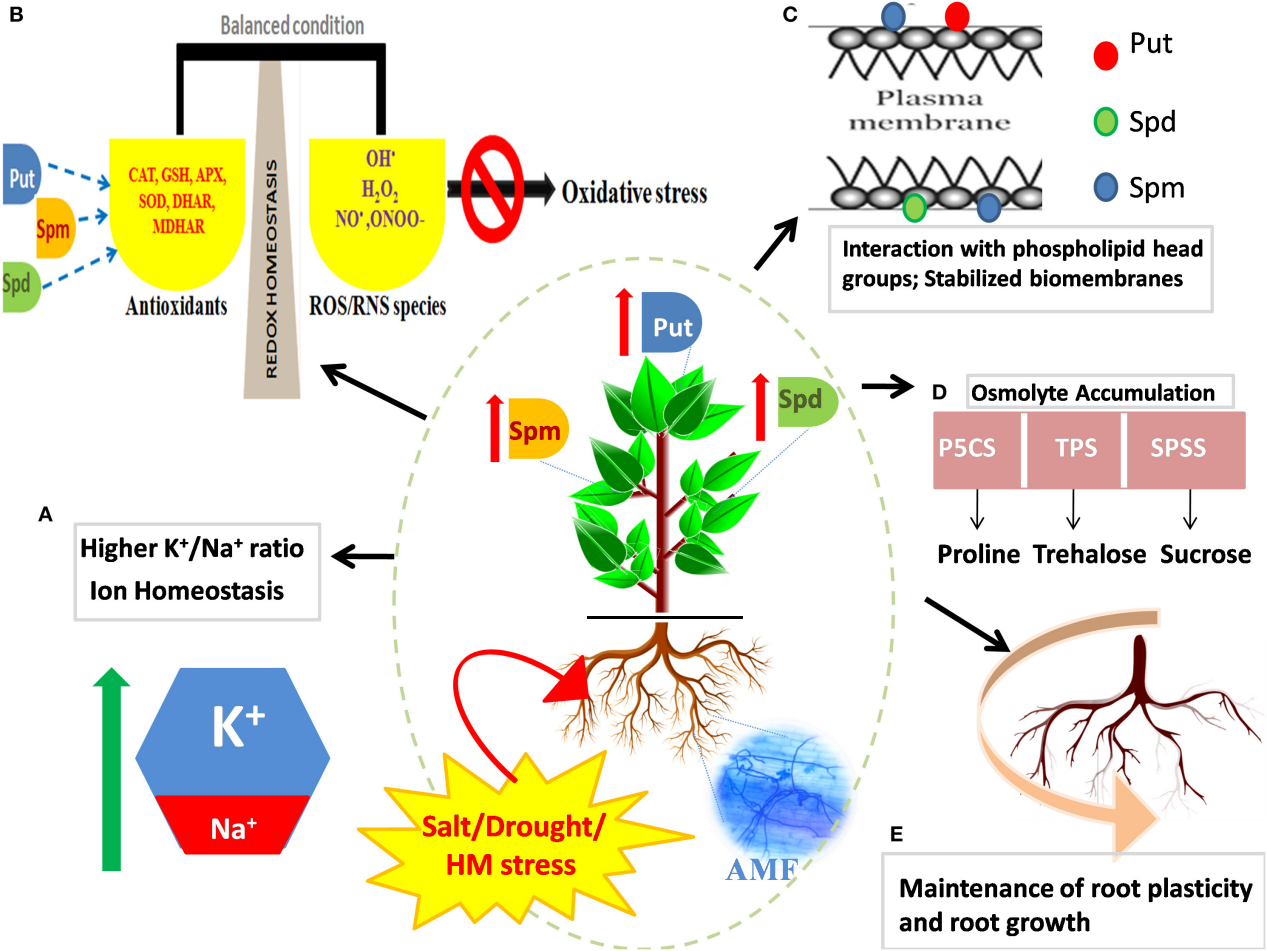
Effect of mycorrhizal symbiosis and abiotic stress on the regulation of PAs and improvement of stress tolerance in plants. In stressed plants, mycorrhizal association refines inherent tolerance of plants by favorable adjustment of the endogenous levels of PAs (Put, Spd, and Spm), the primordial stress molecules. Arbuscular mycorrhizal fungi (AMF) mediated augmented levels of PAs maintain: **(A)** ion homeostasis and upregulated K^+^/Na^+^ ratio, **(B)** redox homeostasis by inducing a battery of enzymatic and non-enzymatic antioxidants, **(C)** the structural and functional framework of biological membranes and macromolecules by interacting with their negatively charged moieties, **(D)** boosted production of protective osmolytes like proline, trehalose, and sugars, and **(E)** root plasticity by regulation of root cell expansion and development of adventitious roots. Several layers of regulation operate to strengthen tolerance mechanism of plants and combat osmotic stress incurred by salinity, drought, or heavy metal (HM) stress.

The close association of PAs with the defense response of plants is ascribed to multiple reasons: (1) transcriptional activity of PA biosynthetic genes [*ADC, ODC, SPDS, SPMS*, and *SAM decarboxylase (SAMDC)*] and catalytic ability of the enzymes that participate in the PA metabolism are reinforced in the presence of stress, (2) induced suppression of PA synthesis by inhibitors such as difluoromethyl ornithine (DFMO), difluoromethyl arginine (DFMA), and cyclohexylamine (CHA) or by knocking down/out PA synthesizing genes, corresponds to a compromised defense response (Urano et al., 2003), (3) abundance of free and conjugated PAs in stress-tolerant cultivars under stressful conditions (Zapata et al., 2004), and (4) overexpression of PA biosynthetic genes results in a concomitant increase in the ability of plants to counter and acclimate to stress (Wen et al., 2008). Most studies till now have reiterated the positive impact of exogenous application of PAs on root/shoot architecture, development, and stress response in plants (for instance, *Panax ginseng;* Parvin et al., 2014, *Zoysia japonica* Steud; Li S. et al., 2016; *Bakraii citrus* seedlings; Khoshbakht et al., 2018, and *Cucumis sativus;* Wu et al., 2018). However, it is increasingly becoming a realm of scientific interest to strategize techniques by which endogenous PA production can be boosted so as to improve the overall development of plant as well as to magnify stress adaptiveness. Transgenic approaches oriented toward overexpressing PA biosynthetic genes and manipulating the intertwined metabolic web in a way that directs the metabolic flux toward the synthesis of stress messengers and scavengers are deemed as efficient techniques. Albeit they are successful, these high-throughput approaches are beyond the financial frontiers of many developing and low–middle-income nations. In this respect, use of AMF to optimize the PA metabolism emerges as an economically effective agronomic option. As aforementioned, AMF ensure the PA homeostasis that further helps in stress mitigation by the maintenance of pH and ion homeostasis, production of osmolytes and improvement of plant water status, ROS scavenging, stability of membrane and photosystem framework, modified expression of stress-responsive genes, regulation of root plasticity (Talaat and Shawky, 2013) (Figure 2), and the regulation of ribosomes, amino acids, and energy metabolism (Li et al., 2018). The mediation of stress tolerance using AMF by reinforcing the PA metabolism as a strategy is discussed in the following sections.

### Salt Stress

High soil salinity directly results in various downstream stresses, viz., hyperionic stress, physiological drought, hyperosmotic stress, and nutrient imbalance (Evelin et al., 2009; Porcel et al., 2012). Several studies have dealt with the association of AMF and the PA metabolism in plants to improve tolerance to salt stress (Sannazzaro et al., 2007; Echeverria et al., 2013; Evelin et al., 2013; Talaat and Shawky, 2013; Abeer et al., 2014).

Sannazzaro et al. (2007) reported an increase in the Spd+Spm/Put ratio in salinity-stressed roots of *Lotus glabra* and ascribed it as a mechanism deployed by AMF to mediate the defense response of plants under unfavorable conditions. Lesser free Put in the leaves could be postulated as a deviation of the metabolic pool either toward the synthesis of higher PAs, Spd, and Spm (that have better protective capacity than Put by virtue of additional NH_2_ groups) or toward proline synthesis (as glutamate is the common precursor of proline and PA, Figure 1). However, the validation of this hypothesis would require exhaustive investigation to elucubrate the relationship among mycorrhization, root architecture, and the balance between primary and secondary metabolism. Additionally, they concluded that both mycorrhizal and non-mycorrhizal plants would be equally stressed since they accumulate similar proline contents. Moreover, no correlation was observed between an increased Spd+Spm/Put ratio and the proline accumulation (Sannazzaro et al., 2007). Contrastingly, the AMF inoculation leads to a significant reduction in DAO and PAO activity in salt-stressed wheat plants that accounted for increased content of free Put, Spd, and Spm, and technically highest Put level, which boosted the fitness of plants to salt stress and influenced the ROS-scavenging antioxidant mechanism in inoculated plants (Talaat and Shawky, 2013). Likewise results were obtained in inoculated *Trigonella foenum-graecum* (Evelin et al., 2013), *Lotus tenuis* (Echeverria et al., 2013), and *Vicia faba* (Abeer et al., 2014) plants, wherein AMF application augmented the free PA titers along with other favorable influences on plant metabolism (Table 1). Premised on a tight correlation of Put synthetases and root architecture, it can be argued that AMF-triggered root Put (and not Spm and Spd) refines root traits, hence fostering better nutrient acquisition and improved drought acclimation, as confirmed by Wu et al. (2012b) in *Citrus tangerine* plants.

**Table 1:** Effects of arbuscular mycorrhizal fungi (AMF) inoculation on modulation of polyamine ratio under abiotic stresses.

**Abiotic stress**	**Plant species**	**AMF Species**	**Effect on polyamine ratio**	**References**
SALT STRESS (0 and 200 mM)	*Lotus glaber*	*Glomus intraradices*	↑(Spd + Spm)/Put ratio; ↑Put in shoots of tolerant plants, ↑Spd and Spm in both tolerant and sensitive plants	Sannazzaro et al., 2007
SALT STRESS (0, 50, 100, and 200 mM)	*Trigonella foenum- graecum*	*G. intraradices*	↑Put, ↑Spd, ↑Spm, Increased content of other osmolytes (total soluble sugars, proline, glycine betaine)	Evelin et al., 2013
SALT STRESS (0 and 150 mM)	*Lotus tenuis*	*G. intraradices*	AM salt-stressed plants showed a higher root PA (Spm, Spd, Put) level than their corresponding non-AM controls; no significant effect of AM under salt stress on total, root, stem, and leaf free PA contents in comparison to control plants	Echeverria et al., 2013
SALT STRESS (4.7 and 9.4 dS m^−1^)	*Triticum aestivum* (Giza 168, Sids 1)	Mixture of *Glomus* spp.	Significant reduction in DAO and PAO activities under salt stressed plants; ↑Put, ↓Spm and Spd in Giza 168, ↓Put, ↑Spm and ↑Spd in Sids 1	Talaat and Shawky, 2013
SALT STRESS (0, 50, and 100 mM)	*Vicia faba*	*Funneliformis mosseae, Rhizophagus intraradices*, and *Claroideoglomus etunicatum*	Significant increase in Put, Spd, Spm levels under all conditions of salinity, with maximum increase in Put concentration (under 100 mM treatment)	Abeer et al., 2014
DROUGHT STRESS (2 cycles of moisture stress)	*Medicago sativa*	*G. fasciculatum*	Higher free polyamine (Spd and Spm) content in symbiotic water-stressed plants; No significant enhancement of polyamine concentration in roots	Goicoechea et al., 1998
DROUGHT STRESS (45–50% max field water capacity)	*Poncirus trifoliata*	*G. mosseae*	↓Put and Spd, ↑Spm content, ↑SPMS activity in drought stressed AM plants	Luo, 2009
DROUGHT STRESS (50% of max. water holding capacity)	*P. trifoliata*	*F. mosseae*	↑Put and Cad, ↓Spd and Spm concentrations, ↑PA catabolic enzyme activity (CuAO; PAO) and Put-synthases (ODC and ADC)	Zhang et al., 2020
DROUGHT STRESS (mild T1 and moderate T2 drought)	*Zea mays*	*R. irregularis*	Significantly, ↓Put content in water stressed (T1 and T2) AM plants, ↑DAO and ↑GABAT activity, ↑GABA accumulation	Hu and Chen, 2020
DROUGHT STRESS (soil WW status (18.08%)	*P. trifoliata*	*F. mosseae*	↑ADC,↑ODC, ↑SPMS, ↑SPDS, ↑DAO, ↑PAO activity. ↑precursor of PA (agmatine, L-ornithine and SAM), ↑Put, ↑Cad, ↓Spd	Zou et al., 2021
HEAVY METAL STRESS (Cd)	*Plantago lanceolata*	*G. fasciculatum*	No significant difference in leaf PA ratio between mycorrhizal and non-mycorrhizal plants. Mycorrhizal roots registered lower (Put/Spd+Spm) ratio	Parádi, 2003
HEAVY METAL STRESS (Cu and Zn)	*Populus alba*	*G. mosseae and G. intraradices*	Induction of *PaSPDS1* and *PaSPDS2* and *PaADC;* ↑free and conjugated PA titers in stressed AM plants; ↑stabilization of heavy metals in soil	Cicatelli et al., 2010
HEAVY METAL STRESS (Pb)	*Calopogonium mucunoides*	*G. etunicatum*	Mycorrhization influenced free amino acid profile in leaves; resulted in depleted arginine content, prioritizing PA synthesis over protein metabolism	Souza et al., 2014

**Figure 3:**
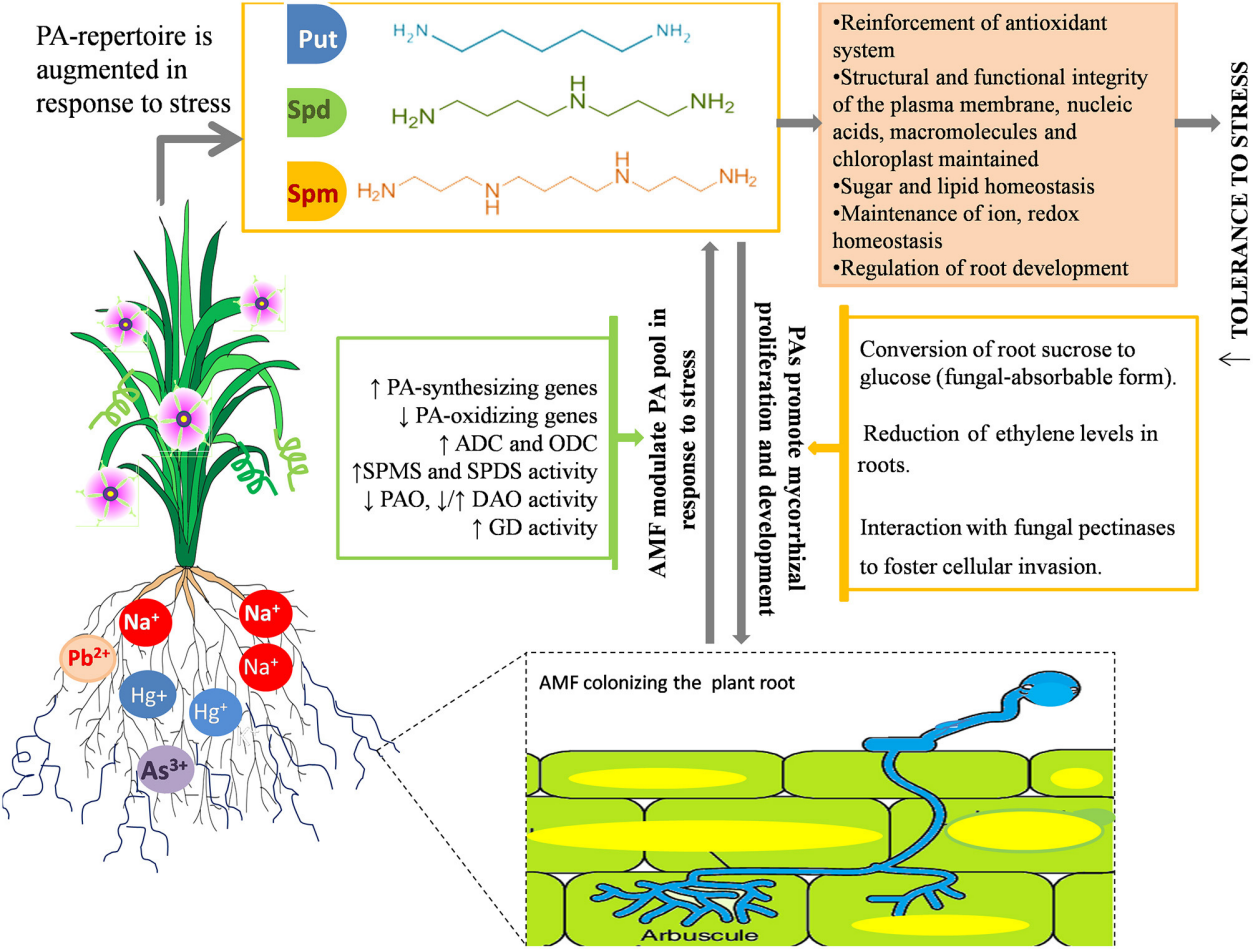
Synergism between AMF and PAs in response to salinity and metal stress. AMF modulates the PA pool at transcriptional and metabolic level by enhancing the expression of PA-synthesizing genes, repressing the genes involved in PA-catabolism and modulating the activity of PA synthesizing enzymes (GD, Glutamate Dehydrogenase; ADC, Arginine Decarboxylase; ODC, Ornithine Decarboxylase; SPMS, Spermine Synthase; SPDS, Spermidine Synthase) and PA-degrading enzymes (DAO-, Diamine Oxidases; PAO, Polyamine Oxidases). Mostly, the synthesis of Spm and Spd is favored over that of putrescine owing to extra stability and protection imparted by the presence of extra amine groups. PAs also foster mycorrhizal growth and development by converting sucrose into fungal-absorbable sugar, glucose; reducing the root ethylene content, which otherwise impedes hyphal development, and interacting with fungal pectinases to boost adhesion and fungal penetration into the host cells. Modulation of PA pool further results in maintenance of a biochemical and structural framework of plants, implicated in enhanced tolerance toward salinity stress.

The synergistic role of PAs and AMF in helping the plant combat stress is also substantiated by mounting evidence that demonstrates how PA and AMF mutually foster each other under stress and concomitantly exert their beneficial effects to make plants more resilient to stress (Figure 3), as the dual administration of AMF and PA is found to be more efficient in maintaining/improving phenotype of the plant under stress than AMF inoculation or PA administration alone (Niemi et al., 2006; Ibrahim et al., 2011; Abdel-Fattah et al., 2013). A comprehensive conclusion can be squeezed out from all the investigations carried out hitherto; there exists a tightly regulated coordination between AMF and the PA metabolism that is committed to refine physiological plasticity of plants and resilience to assure its survival under unfavorable environments. These implications may be of practical importance in realizing the economically feasible, yet efficient, development of salt-tolerant crops.

### Drought Stress

Notwithstanding the precise role of PAs in abiotic stress tolerance and the intimate mechanisms of such an effect are uncertain, there has been mounting evidence evincing the role of PAs in drought-stressed plants. Several studies corroborate the fact that the PA reservoir of plants corresponds to drought stress tolerance. For instance, Nahar et al. (2016) reported in *Vigna radiata* the Spm-mediated upregulation of antioxidant genes and decreased methylglyoxal toxicity by the activity of refined glyoxalase system. This system plays a critical role in counteracting oxidative stress by recycling reduced GSH, which is entrapped by methylglyoxal, consequently maintaining GSH homeostasis. Higher PA levels were recorded in water-stressed wheat, as postulated to be responsible for drought tolerance and grain filling (Liu et al., 2016). PAs moderate the evolution rate of ethylene (antagonistic behavior), which might have accounted to better grain filling in wheat plants. In a pioneering study by Hura et al. (2015), drought stress tolerance in triticale during late developmental stages (from tillering to anthesis) was associated with specific accumulation of cell membrane-bound PAs and decreased content of free PAs. This could be accredited to the role of PAs in the reinforcement of plant cell wall under stress. Besides, PAs are also capable of regulating stomatal movements under drought stress conditions by encumbering inward rectifying K^+^ channels (KIRC) (Liu et al., 2000) (Figure 2). The binding of Put ions to the KIRC site propels the outward displacement of K^+^ ions and further retards K^+^ movement across the channel (Chen et al., 2020). PAs are significant targets in AMF-mediated drought stress regulation. However, inconsistency in PA accrual patterns has been observed in AM plants in response to water deficit. The first pioneering study in reference to this was conducted by Goicoechea et al. (1998) wherein the inoculation of water-stressed alfalfa plants with *Glomus fasciculatum* resulted in the following: (1) an increment of free Spm and Spd levels, which might serve as a nitrogen reserve that can be used to fulfill immediate energy demands of the plants soon after it recuperates from stress and (2) a higher enhancement of PA concentration in leaves than that in roots, which might be due to a high transpiration rate observed in drought-stressed plants that fostered a long-distance transport of PAs from root to shoot *via* transpiration stream. Also, the free Spd contents of stressed AM plants were closely correlated with proline levels. In contrast to that, Zhang et al. (2020) observed that besides boosting growth traits and AQP expression in *Poncirus trifoliata* plants, *Funneliformis mosseae* augmented the cellular concentration of specifically two diamines, Put and cadaverine (Cad), and diminuted the concentrations of root Spd and Spm under water-deficit conditions. This is ascribed to a concomitant increase in the activity of Put-synthases (*ADC* and *ODC*) and Put-oxidases (*PtPAO1, PtPAO2*, and *PtPAO3*) that convert Spd back to Spm and further to Put, by successive deamination (Mo et al., 2015). The upregulation of *PAO* expression is important to stimulate stress responsiveness in plants probably because it results in the production of H_2_O_2_, which, when present in moderate amounts, serves as a ligand in stress signaling (Sequera-Mutiozabal et al., 2017).

Another study on *P. trifoliata* inoculated with *Glomus mosseae* and subjected to drought stress exhibited substantially lowered concentrations of Put and Spd and significantly elevated levels of Spm in inoculated (vs. non-inoculated) plants, thereby advocating the essential role of Spm in trifoliate orange (Luo, 2009). The increased titers of free Spm could be explained by the higher activity of the *SPMS* gene and drought-spurred reduction of its metabolic precursors, Put and Spd. Better protective role of higher PAs (owing to the additional amine groups) could also offer a good explanation for the higher Spd concentration obtained. The results of an experiment on maize seedlings exposed to water-deficit conditions suggested that Put oxidation into GABA [by the activity of copper amine oxidase (CuAO)] was the target pathway in AM-facilitated regulation of drought tolerance (Hu and Chen, 2020). GABA shunt is vital for C/N homeostasis in plants (Shelp et al., 2012) and drought acclimation (Mekonnen et al., 2016). Moreover, the activity of GABA transaminase (GABAT) was significantly high in AM roots under drought. GABAT converts GABA into a subsequent substrate, succinate, which is consumed in the tricarboxylic acid cycle (TCA) cycle to synthesize carbohydrates. In a recent study by Zou et al. (2021), AMF-regulated PA homeostasis in the roots of *P. trifoliata* plants was found to be associated with enhanced tolerance against soil moisture deficit stress (SMDS). Mycorrhizal plants exhibited a significantly higher concentration of PA precursors, L-ornithine, agmatine, and SAM, increased Put and Cad, and reduced Spd content. In response to SMDS, AMF colonization augmented the activities of PA-synthetic (ADC, ODC, SPMS, and SPDS) and catabolizing (PAO and DAO) enzymes with much less degree of damage to the membrane and lower generation of ROS *vis-à-vis* non-mycorrhizal plants (Table 1). It can be speculated from the studies so far that the accrual pattern of PAs may totally vary depending on the type of host plant, the interacting AMF partner, the degree, duration, and nature of stress, and most importantly, the developmental stage and tissue of the plant under investigation; all these factors might account for the variation in free and/or conjugated PA pool observed in different plants.

The inherent and AMF-facilitated modulation of PA titers may behave either as an indicator or a harbinger (to communicate the perceived environmental cues) to modify spatio-temporal response of plants to stress (Rangan et al., 2014). That being said, further studies should be dedicated to unraveling the molecular signaling pathways of AM-induced regulation of these metabolic hubs. With the advent of forward and reverse genetic approaches, the functional essence of PAs in stress acclimation has been elucidated to some extent. However, a straight cause-and-effect relationship between the accumulation of PAs and drought stress is not firmly established yet (Pál et al., 2015). The knowledge acquired so far about the AMF-mediated modulation of PAs has constructed a strong case for future studies earmarked for vigilant analyses of fungal genes contributing to the modulation of PA pool upon stress exposure. High-throughput investigations involving transcriptomics, metabolomics, and microarray approaches along with a careful analysis of coordinating partners of PA metabolic enzymes will be a great deal of help in better understanding the mechanism of stress tolerance/resistance and whether it involves PA-induced epigenetic changes in the plant cells.

### Heavy Metal Stress

In the process of phytoremediation, many plant-associated microbes reinforce the metal detoxification capacity of the plants (microbe-assisted phytoremediation). HM-contaminated soils can be redeemed and revegetated by using AMF that can potentially expedite the phytostabilization and phytoextraction process (Khalid et al., 2021). The potential of AMF as an ameliorator of HM toxicity has been well-proved and promoted (Christie et al., 2004; Bai et al., 2008; Merlos et al., 2016; Sharma et al., 2017; Gupta et al., 2021). Fungus-assisted phytoremediation is deemed as the most befitting and eco-friendly technology for the redemption of HM polluted soil (Khalid et al., 2021). Among various recognized mechanisms deployed by AMF for the prevention of HM uptake by plants (metal immobilization, modulation of metal transporter activity, and production of organic acids and glomalin), the regulation of the PA metabolism under metal stress is inconsiderably explored (Ezawa et al., 2002; Smith and Read, 2008; Chen et al., 2012; Tajti et al., 2018). PAs can stimulate metal chelation, boost antioxidant defense, maintain hormone homeostasis, and ensure membrane stability (Tajti et al., 2018). The stress mitigatory roles of PAs explain their inherent regulation during metal stress.

In cadmium-treated (Cd) and copper-treated (Cu) wheat leaf segments, a respective increase and decrease in Put and Spm concentration were obtained (Groppa et al., 2007). Such results might be attributed to the following: (1) accelerated PA (Spd and Spm) degradation into Put by higher PAO activity or (2) higher SAM flux toward ethylene metabolism, formation of which is detrimental to higher PAs. Resultantly, the reduction of Put to Spd and Spm was hampered in spite of Put availability. Similar fluctuations in PA titers have been observed in Cu-stressed rice (Lin and Kao, 1999), Cd-stressed *Potamogeton crispus* L. (Yang et al., 2010), and chromium- (Cr) stressed *P. trifoliata* (Shahid et al., 2018), etc. Besides, exogenous feeding of PAs also refines the resistance of plants to HM stress (Rady and Hemida, 2015; Nahar et al., 2016; Taie et al., 2019). Garg and Saroy (2020) demonstrated that the combined application of AMF (*Rhizoglomus intraradices*) with PAs furnished a remarkable improvement in biomass (root and shoot) yield of plants, nutrient acquisition, ureids and trehalose accumulation, and nodulation potential in *Cajanus cajan* under nickel (Ni) stress.

A plethora of reports on both AMF-mediated and PA-mediated increase in oxidative defense strategy, such as the reinforced activity of enzymes participating in AsA-GSH cycle, contributing to stress tolerance in plants, exist in the literature (to cite a few, Garg and Bhandari, 2016, Tseng et al., 2013; Liu et al., 2017; Hashem et al., 2018). In a recent study, Saroy and Garg (2021) analyzed the effectiveness of AMF and PAs in modulating the AsA-GSH cycle in two genotypes of *C. cajan* varying in their Ni tolerance. They found out that exogenous co-supplementation of PAs (mainly Put) and *R. intraradices* contributed the most in upsurge in the activity of enzymes of AsA and GSH pool [ascorbate peroxidase (APOX), monodehydroascorbate reductase (MDHAR), dehydroascorbate reductase (DHAR), glutathione reductase (GR)] and also improved GSH/glutathione oxidized (GSSH) ratio, consequently providing tolerance against the HM-induced oxidative damage in pigeon pea genotypes. Beyond the repressive mechanisms of PAs on HM uptake, the latter also boost the production of phytochelatins (PCs), which bind, transport, and sequester the metal ions into the vacuole (Pál et al., 2017); possibly because they contribute to GSH pool, which is the building block of PCs. Higher PAs behave as stronger chelators or amplify chelation mechanisms (Nahar et al., 2016). Furthermore, PAs can also block cation channels in the sequence, Spm ^4+^> Spm^3+^> Put^2+^, hence, they succor the sequestration of surplus metal ions during stress (Liu et al., 2014).

To what extent the innate stress resistance mechanisms of the host are persuaded by mycorrhizal symbiosis still remains ambiguous. Although mycorrhizal mediation of metallothionein (*MT*), *PC* and HM tolerance (*HMT*) genes under HM stress have been unequivocally established, the “buffer effect” of AMF in improving the HM resistance of AM plants by orchestrating the PA metabolism has been very modestly studied and reported. Parádi (2003) studied the influence of *Glomus intraradices* on free PA content and ratios in Cd-exposed *Daucus carota* L. plants. AM plants did not register any significant change in the PA ratio unlike non-AM counterparts that showed reduced Spd and Put content. This could be due to the “equalizing effect” of AMF upon Cd exposure against the alteration in PA titers widely reported in various stress conditions. In poplar plants (clone AL35) grown in Cu and Zn polluted soil, inoculation with *G. mosseae (Gm)* and *G. intraradices (Gi)* provoked an overall increment in free and conjugated PAs by the induction of *PaSPDS2* and *PaADC* expression (Cicatelli et al., 2010, 2014). Contrary to the first sampling stage (S1), *PaADC* expression was downregulated at second sampling stage (S2) in mycorrhizal plants under stress. In the presence of metal stress, both *PaSPDS1* and *PaSPDS2* were upregulated in *Gi*-inoculated poplar plants. At S2, both SPDS transcripts were induced by the inoculation with AMF on both non-stressed and stressed soil. At S1, on non-polluted soil, free PA levels were strongly reduced in the presence of AMF, while those of conjugated PAs were significantly higher in AM plants. On polluted soil, free Spd titers were significantly higher in the presence of AMF, especially *Gi*; conjugated Spd and Spm titers were also dramatically enhanced (up to 5-fold) relative to uninoculated controls, but only in *Gi*-inoculated plants (Cicatelli et al., 2010, 2014). Amelioration of lead (Pb) toxicity in mycorrhized *Calopogonium mucunoides* was related to the alteration in amino acid profile, deviating metabolic flux toward PA synthesis from protein synthesis (Souza et al., 2014) (Table 1). Taken together, these results advocate that stress amelioration is driven from AMF-mediated regulation of these protective molecules under the combined effect of toxic metals and AMF.

Research on AMF-regulated PA metabolism and function during metal stress is in its infancy; hence, countless efforts are required to unravel the molecular dialog of AMF and PA and their protective role in HM stress tolerance. Employment of “-omics” approaches is needed to amplify AMF-mediated PA actions toward HM toxicity. Also, the effect of co-inoculation of AMF with growth-promoting bacteria in reversing metal toxicity should be subjected to thorough scrutiny to fathom the potential of synergistic interactions of plant-associated microbes in the mediation of crop abiotic stress tolerance.

### Impact of Polyamines on Mycorrhizal Colonization

The extent of mycorrhizal colonization, spore germination, and hyphal growth and propagation are also affected by various stressors (Wu et al., 2010; Shekoofeh et al., 2012). In that respect, PAs have been reported to, in turn, enhance the AM symbiosis by actively participating in signaling episodes of plant–fungus interaction (El Ghachtouli et al., 1995). This positive correlation between mycorrhizal colonization and PAs is validated by several studies. Based on their type and method of administration (soil drench or foliar application), PAs exerted their effectiveness on mycorrhizal (*G. intraradices*) development and concomitantly improved the quantity and quality of corm, and enhanced flower parameters in *Freesia hybrida* (Rezvanypour et al., 2015). The application of PAs in grape plants yielded beneficial effects on the infection rate, spore number, and hyphal growth of *Gigaspora margarita*, which are significantly reduced by the application of a PA biosynthetic inhibitor, methylglyoxal bis-guanylhydrazone (Yong et al., 2003). These results have made the stimulatory effects of PAs on the symbiotic efficiency of AMF manifestable and apparently directed toward the role of PAs as potential “regulatory factors” in the mycorrhizal association. Wu et al. (2012b) reported that 14 weeks of exogenous PA application on *C. tangerine* inoculated with *Paraglomus occultum* resulted in accelerated mycorrhizal colonization, a greater number of infectious propagules (vesicles and arbuscules), an enhanced mycorrhizal status, and a higher root glucose concentration, suggesting that PAs probably facilitate the transformation of root sucrose to glucose (usable form of carbohydrate for the fungus) to promote mycorrhizal proliferation and development. The PA-mediated stimulation of root colonization can be attributed to various reasons: (1) PAs directly interact with fungal pectinases and boost adhesion, fungal penetration, and growth into the cell wall of plants (Nogales et al., 2009), (2) PAs reduce the ethylene levels in roots, which otherwise inhibit root colonization (Foo et al., 2016; Mattoo and White, 2018), and (3) AMF proliferation and development begins with chromatin decondensation of the host nucleus, corresponding to higher transcriptional activity (Berta et al., 1990), essential to which are PAs as they stabilize RNA against RNase activity (Serafini-Fracassini et al., 1984). Thus, PAs function as regulatory molecules in plant–AMF interactions (El Ghachtouli et al., 1995) (Figure 3).

## AQUAPORINS: Types and Background

One of the crucial parameters that govern the development and stages of growth in plants is the stringent regulation of transcellular water movement, conduction, and mobilization. Diffusion of water across cells is facilitated by AQPs, the members of major intrinsic proteins (MIPs) family, that serve as a nexus between and within the cells, passively conducting water, gases, and solutes. Multiple isoforms of AQPs are found in plants, the fact that is reflective of a high degree of compartmentalization of cells, selectivity and size specificity of transport, and regulation properties that fine-tune water transport (Kapilan et al., 2018). As per the amino acid sequence homology, MIPs can be categorized into four major subgroups: (1) plasma membrane intrinsic proteins (PIPs) constitute the largest subfamily of MIPs and sit predominantly in the plasma membrane of the cells localized in the plant organs marked by high water fluxes, such as guard cells and root cells; (2) tonoplast intrinsic proteins (TIPs) chiefly reside in the vacuolar membrane serving as conduits for the transportation of water, small solutes, metabolites, and gases, thereby contributing to rapid osmotic homeostasis of the cytosol and maintenance of the cellular hydrostatic pressure; (3) nodulin-26 like intrinsic proteins (NIPs) are the aqua-glyceroporins found localized in the peribacterial membranes of the root nodules, hence being supposed to be presumably involved in the exchange of metabolites (glycerol and water, chiefly) betwixt the host plant and the N_2_-fixing bacterial symbionts; and (4) small basic intrinsic proteins (SIPs) are localized in the membrane of endoplasmic reticulum (ER); however, their precise physiological functionality is indeterminate (Ishikawa et al., 2005).

Expression and activity of AQPs are regulated by several processes, such as gating, which deals with the phosphorylation and dephosphorylation of AQPs, heterotetramerization, divalent cations, hormones, and even ROS generated during abiotic stresses (Kapilan et al., 2018). Tight regulation of these molecular gears can enable efficient regulation of water conduction under stress. Owing to the isoform multiplicity of AQPs and distinct expression patterns under stress (it may reduce, increase, or may remain unchanged), the discrete and integrated functionality of AQPs under diverse physiological conditions under stress remains elusive. The regulation of water conduction by AQPs under a variety of abiotic stresses depends on multiple parameters: the nature, duration and intensity of stress, conditions of plant growth, developmental stage, the type of tissue expressing AQP genes, and the type/isoform of AQP being expressed (Siemens and Zwiazek, 2004). There is a considerable dearth of such studies in the literature that directly integrate the relationship among the two biomolecules (PA and AQP) and AMF *per se*. However, it can be speculated that PA homeostasis has some roles to play in regulating the distribution and abundance of AQP proteins by virtue of which plant–water relations are maintained (Tailor and Bhatla, 2021). The authors studied the effect of potent PA inhibitors (DFMA and DFMO) on the expression of two major AQP families: PIP2 and TIP1 in salt-stressed *Helianthus annuus* L. seedlings. The seedling that was raised in 500 μM of DFMO and DFMA exhibited a significant root extension, irrespective of the salt-stressed imposed, and resulted in further exacerbating the decrease in relative water content (RWC) in roots and cotyledons. This was found to be correlated with augmented levels of PIP2 and TIP1 proteins in the roots but not in the cotyledons, of the seedling (Tailor and Bhatla, 2021). This arena of study integrated with the role of AMF colonization in further modulating the two biomolecules can offer a good research question to investigate. On this account, it can be deduced, *a priori*, that AMF-regulated PA homeostasis that accounts for improved adaptation of plants to water-deficit conditions (Hu and Chen, 2020; Hu et al., 2020; Zhang et al., 2020) could be correlated to an extent as a function of modulation of the abundance of AQP subfamilies. However, no direct correlation between PAs and AQPs has been established hitherto.

## AMF-Mediated Regulation of aquaporins Under Stress

Complete establishment of AM symbiosis in plants needs extensive morphological and molecular reprogramming with most of the morphological alterations concerned with changes in membrane system of vacuole and cytoplasm. Since AM symbiosis potentially alters root hydraulic conductivity in plants, the fact that it regulates AQP gene expression and protein abundance seems more convincing. The first wave of an attempt to characterize AMF-mediated changes in the AQP profile rose by Roussel et al. (1997), who reported mycorrhiza-induced *TIP* expressions in *Petroselinum crispum*, followed by that of Krajinski et al. (2000), who conducted the experiment along similar lines in *Medicago truncatula* and completely credited the changes of *MtAQP1* expression profile to AMF colonization. They proposed that mycorrhiza-induced heightened expression of *MtTIP* is crucial to optimize water conduction after the changes that commence in plants on symbiosis establishment. Arbuscule-containing root cortical cells may register an altered ratio of cytoplasmic content and vacuolar space, due to which, changes in the tonoplast may be required to buffer the osmotic instability in the cytoplasm. Higher *MtTIP* expression could offer a way of redressing the reduced tonoplast water permeability of the highly compartmented vacuoles in the cells harboring fungal symbionts. Analogously, *Medicago* plants inoculated with *G. mosseae* showed an apparently enhanced expression of *MtPIP2;1* and *MtNIP1* while transcript abundance of other isoforms were uninfluenced by mycorrhization (Uehlein et al., 2007) (Table 2). The peri-arbuscular membrane (PAM) surrounding the arbuscules is a site of transmembrane water and solute transport across the plant–fungus interface. The strong induction of AQP expression during mycorrhization might be indicative of the physiological alterations, i.e., the optimization of water and solute transport system in the roots (the site of plant–fungus interaction), as symbiotic exchange occurs through AQPs localized in the PAM. AQPs furnish a very low-resistance transcellular water conduction pathway through the membrane, which seems to be predominantly managed by TIPs and PIPs that are the central regulators of the conduction pathway (Maurel, 2007). Many AMF-regulated AQPs conduct solutes such as glycerol, urea, and H_2_O_2_ along with water, all of which are vital to the physiological performance of plants (Bárzana et al., 2014). Apart from these roles, AQPs also exert a potential influence on redox events in plants under unfavorable conditions. Additionally, since AQPs can be gated through reversible phosphorylation and dephosphorylation, this offers a way to control the water fluxes and movement dynamics across the cellular and vacuolar membranes.

**Table 2:** Summary of studies on effects of AM symbiosis on aquaporin (AQP) gene expression under non-stressed, water-deficit (salt and drought stress), and heavy metal (HM) stress conditions. The consequences of AMF-mediated AQP regulation on physiology of stressed plants are also included.

**Abiotic conditions**	**Plant species**	**AMF Species**	**AMF-mediated effects on Aquaporin gene expression**	**Consequences on physiology of AM Plants**	**References**
No Stress	*Petroselinum crispum*	*Glomus fasciculatum*	↑*PcTIP*	Plant water status not measured	Roussel et al., 1997
	*Medicago truncatula*	*G. mosseae*	↑*MtTIP*	Plant water status not studied	Krajinski et al., 2000
	*Poplar tremula × tremuloides*	*G. mosseae*	↑*PttPIP1.1*, ↑*PttPIP2.3*, ↑*PttPIP2.5*	Increased L_o_ in AM plants	Marjanović et al., 2005
	*Medicago truncatula*	Unpublished data	↑*MtPIP2;1* and ↑*MtNIP1*	Plant water status not taken into account	Uehlein et al., 2007
Salt stress	*Lycopersicum esculentum*	*G. geosporum and G. intraradices*	↓*LePIP1* and ↓*LeTIP* =*LePIP2*	Plant water status analysis not conducted	Ouziad et al., 2006
	*Phaseolus vulgaris*	*G. intraradices*	↑*PvPIP1.1*,=*PvPIP1.3*, ↑*PvPIP2.1*,↓*PvPIP1;2*	Increase in J_v_, L_o_, and RWC	Aroca et al., 2007
	*Lactuca sativa*	*G. intraradices*	↑*LsPIP1* and =*LsPIP2*	Increased RWC, lower ABA accumulation in roots, lower proline content	Jahromi et al., 2008
	*Robinia pseudoacacia*	*Rhizophagus irregularis*	**Roots:** ↑*RpPIP1;3*, ↑*RpPIP2;1*, ↑*RpTIP1;1* ↓*RpTIP1;3* **Leaves**: ↓*RpPIP2;1* and ↓*RpTIP1;3* ↑*RpPIP1;1* and ↑*RpPIP1;3*	Increase in the RWC at 200 mM NaCl by 9%., increased WST, improved Pn by 106% at 100 mM NaCl and by 81% at 200 mM NaCl, Higher WUE, Lower Ci, higher Gs than non-AM plants	Chen et al., 2017
	*Poncirus trifoliata*	*Paraglomus occultum*	↓*PtTIP1;1*, ↓*PtTIP1;2* and ↓*PtTIP1;3* =*PtTIP2;1*, =*PtTIP2;2* ↑↑*PtTIP4;1* (no salt)	Spurred root water absorption, accelerated	Ding et al., 2020
	*Lactuca sativa*	*F. mosseae and Claroideoglomus lamellosum*	↑↑*PtTIP5;1* (salt stress) No effect on PIP1, ↑PIP2 (↑PIP2A, ↑PIP2B, ↑PIP2C) abundance	Leaf Ψ by 9.14%, significantly higher RWC	Santander et al., 2021
Drought stress	*Glycine max*, *Lactuca sativa*	*R. intraradices*	↓*GmPIP1*, ↓*GmPIP2* ↓*LsPIP1*, ↓*LsPIP2*	Higher leaf Ψ and RWC	Porcel et al., 2006
	*Phaseolus vulgaris*	*G. mosseae*	↓*PvPIP1;1*, ↓*PvPIP1;2*, ↓*PvPIP1;3* ↓*PvPIP2;1*	Increased RWC and J_v_	Aroca et al., 2007
	*Zea mays*	*R. intraradices* Isolate BEG 121	↑*ZmPIP1;1*, ↑*ZmPIP1;2* ↑*ZmPIP2;5*, ↑*ZmPIP2;6* ↓*ZmPIP2;2*	Application of exogenous ABA enhanced J_v_ and L_o_ in AM and non-AM plants, regardless of the water regime	Ruiz-Lozano et al., 2009
	*Z. mays*	*R. intraradices*	**Short term drought:**	Increased J_v_ and L_o_ values	Bárzana et al., 2014
			↑*ZmPIP1;1*,↑*ZmPIP1;2*	Increased L_h_ values under	
			↑*ZmPIP1;3*,↑*ZmPIP1;4*	drought	
			↑*ZmPIP1;6*,↑*ZmPIP2;2*		
			↑*ZmPIP2;4*,↑*ZmPIP1;1*		
			↑*ZmPIP1;2*,		
			↑*ZmTIP1;1*,↑*ZmTIP1;2*		
			↓*ZmNIP2;1*,↓*ZmNIP2;2*		
			**Sustained drought**: ↓*ZmPIP1;1*, ↓*ZmPIP1;1* *↓ZmPIP1;3/ZmPIP1;4*, ↓*ZmPIP2;2*, ↓*ZmPIP2;4* ↓*ZmNIP2;1*,↓*ZmNIP2;2* ↓*ZmTIP1;1*,↓*ZmTIP1;2*	Decreased J_v_ and L_o_ values, reduced sap flow, decreased Si uptake, decreased B uptake, enhanced RLWC, Leaf Ψ, and ABA level in roots and better plant growth performance	
	*Robinia*	*R. irregularis*	**Roots**: ↓*RpTIP1;1* ↓*RpPIP1;3*	Higher dry mass and lower WSD and	He et al., 2016
	*pseudoacacia*		**Stem:** ↑*RpTIP1;1*, ↑*RpTIP2;1*, ↑*RpPIP2;1*, ↓*RpPIP1;1*, ↓*RpPIP1;3*	electrolyte leakage, increased leaf Pn and Gs	
			**Leaves:** ↑*RpTIP2;1*, ↑*RpPIP2;1* ↓*RpTIP1;3*, ↓*RpPIP1;3*		
	*Poncirus trifoliata*	*G. mosseae*	↑*PtTIP1;2*, ↑*PtTIP1;3*, ↑*PtTIP4;1* ↓*PtTIP2;1* and ↓*PtTIP5;1; PtTIP1;1* and *PtTIP2;2* unaffected	Significantly enhanced leaf RWC, Leaf Ψ, and plant growth performance (plant height, stem diameter, leaf number, and biomass), elevated root ABA levels	Jia-Dong et al., 2019
	*Z. mays*	*R. irregularis*	↑*ZmPIP2;1*, ↑*ZmPIP2;6*	Enhanced Pn, gs, Ci, and water permeability of mycorrhizal plants	Quiroga et al., 2019a
	*Z. mays*	*F. mosseae*	↓*PtPIPs*, unaltered *PtTIPs*, ↓↓*PtNIP1;1*, ↓↓*PtNIP5;1*, ↓↓*PtNIP6;1*	Enhanced Pn, gs, E, leaf Ψ, and RWC; lower Lt. Increased plant growth performance of AM plants	Zou et al., 2019
Heavy Metal Stress (low, moderate and high Cu stress)	*Salix purpurea L*.	*R. irregularis*	↑*TIP2;2* in roots of inoculated plants, ↑*PIP1;2* in low-Cu treatment samplings, *PIP2;2* steady among all Cu-treatments	Increased L_o_, moderated K_L_, increased antioxidant capacity (↑SOD and ↑APX)	Almeida-Rodríguez et al., 2016

*J_v_, sap flow rate; L_h_, hydrostatic root hydraulic conductance; RWC, relative water content; ABA, abscisic acid; L_o_, root hydraulic conductance; Lt, leaf temperature; Si, silicon; B, boron; RLWC, relative leaf water content; Ci, intracellular CO_2_ concentration; Pn, net photosynthetic rate; WSD, water saturation deficit; Gs, stomatal conductance; WUE, water-use efficiency; Ψ, water potential; SOD, superoxide dismutase; APX, ascorbate peroxidase; K_L_, leaf-specific conductivity*.

### Salt Stress

The strategic effect of mycorrhization on water acquisition and transport in salt-stressed and non-stressed plants strongly suggests its impact on AQP channels that mediate water transport in plants (Chen et al., 2017). The expression profile of *AQPs* in mycorrhizal plants experiencing salinity stress has revealed strikingly different results in different studies. Ouziad et al. (2006) reported that the transcript levels of *LePIP1* and *LeTIP* exhibited a significant reduction in the roots and a slight upregulation in the leaves of the salt-stressed tomato colonized with *Glomus* spp. The tissue-specific expression suggests that fungi might acquire the function of water mobilization and transportation from roots to shoots, rather than the water uptake by roots from the soil, as the latter would allow the ingression of toxic Na^+^ and Cl^−^ ions into the roots along with the inflow of water; as few AQPs are reported to have Na^+^ conduction properties, like *AtPIP2;1* and *AtPIP2;2* of *Arabidopsis* (Kourghi et al., 2017). In another study, the transcript levels of *LsPIP1* and *LsPIP2* were inhibited by mycorrhization (*G. intraradices*) in non-stressed *Lactuca sativa* plants; however, the expression of *LsPIP1* got enhanced with 100 mM high-salinity NaCl dose, while no stark difference in that of *LsPIP2* was notable between AM and non-AM plants (Jahromi et al., 2008).

The dynamism of AQP responses depends on the strain of AMF and the host type, the intrinsic nature and mode of the osmotic stress applied, and the isoform of AQP, adding layers of complexity to the AMF-mediated regulation of AQP expression (Jahromi et al., 2008; Ruiz-Lozano and Aroca, 2010). Four *PIP* genes analyzed in *Phaseolus vulgaris* plants colonized with *G. intraradices*, and subjected to salinity, low temperature, or drought stress, revealed contrasting results (Aroca et al., 2007). Three of the genes investigated exhibited differential regulation by mycorrhization under each stress regime. Salinity stress resulted in the upregulation of all the *PIP* genes in both sets of plants (AM and non-AM), with a considerable enhancement in AM plants (Table 2). Such a kind of differential AQP expression registered under each stress based on the presence of AMF may be indicative of the following: (1) difference at the level of regulation under the specified set of stresses, (2) different functions performed by each PIP isoform analyzed under each stress examined, and (3) substitution/compensation of the roles performed by plant AQPs by that of fungal AQPs depending on the particular stress foisted. However, to confirm the latter hypothesis, a simultaneous expression profile of fungal AQPs under different stress episodes needs to be generated.

Chen et al. (2017) detected an upregulation of four AQP genes, *RpPIP1;1, RpPIP1;3, RpPIP2;1*, and *RpTIP2;1*, in all the mycorrhizal *Robinia pseudoacacia* plants at high-salinity dosage (200 mM of NaCl). In this experiment, AM plants were found to exhibit lower values of intracellular (carbon dioxide) CO_2_ concentration (Ci) under all doses of salinity. Higher Ci value reflects demolition of the photosynthetic apparatus and passivation of enzymes involved in CO_2_ fixation that results in reduced CO_2_ assimilation (Sheng et al., 2008). Besides, AM plants also experienced less reduction in Gs (stomatal conductance) values under salinity, which indicated better water status of mycorrhized plants, enabled by “maintained” CO_2_ diffusion through stomata by the regulation of leaf AQPs. Likewise, in another recent study on *P. trifoliata*, inoculation with *P. occultum* resulted in differential expression of TIP isoforms and spurred water absorption (↑RWC) and accelerated the leaf Ψ (water potential) by 9.14% (Ding et al., 2020). In *L. sativa* [two cultivars, Grand Rapids (GR) and Lollo Bionda (LB)] inoculated with a consortium of *F. mosseae* and *Claroideoglomus lamellosum*, no alteration in PIP1 abundance was observed, which the authors attribute to the conservative nature of these proteins. In salt-stressed AM cultivars, increased phosphorylation levels of PIP2A, PIP2B, and PIP2C were observed compared to the non-inoculated counterparts. Also, an increased level of PIP2 proteins was observed in the membrane of inoculated GR plants as opposed to non-inoculated ones subjected to salt stress (Santander et al., 2021) (Table 2). All the studies analyzed, hitherto, are congruent with the findings of Valot et al. (2005) that the inoculation of a plant (*M. truncatula*) with mycorrhiza (*G. intraradices*) differentially regulates several plasma membrane proteins, some of them are inhibited while some of them are induced.

There are currently two schools of thought that explain the diverse expression of AQPs registered in response to AMF under desiccation stress. The first one is premised on the induced expression of AQPs under dehydration stress, which is plausibly explained as a mechanism to boost membrane permeability and facilitate the water uptake and conduction in planta. The second is premised on the fact that the expression of AQPs is inhibited in plants experiencing dehydration stress; this might be an efficient strategy to facilitate water conservation by decreasing membrane permeability to water as well an endeavor of the plant to conserve metabolic energy under stressful conditions (Sonah et al., 2017).

### Drought Stress

**Figure 4:**
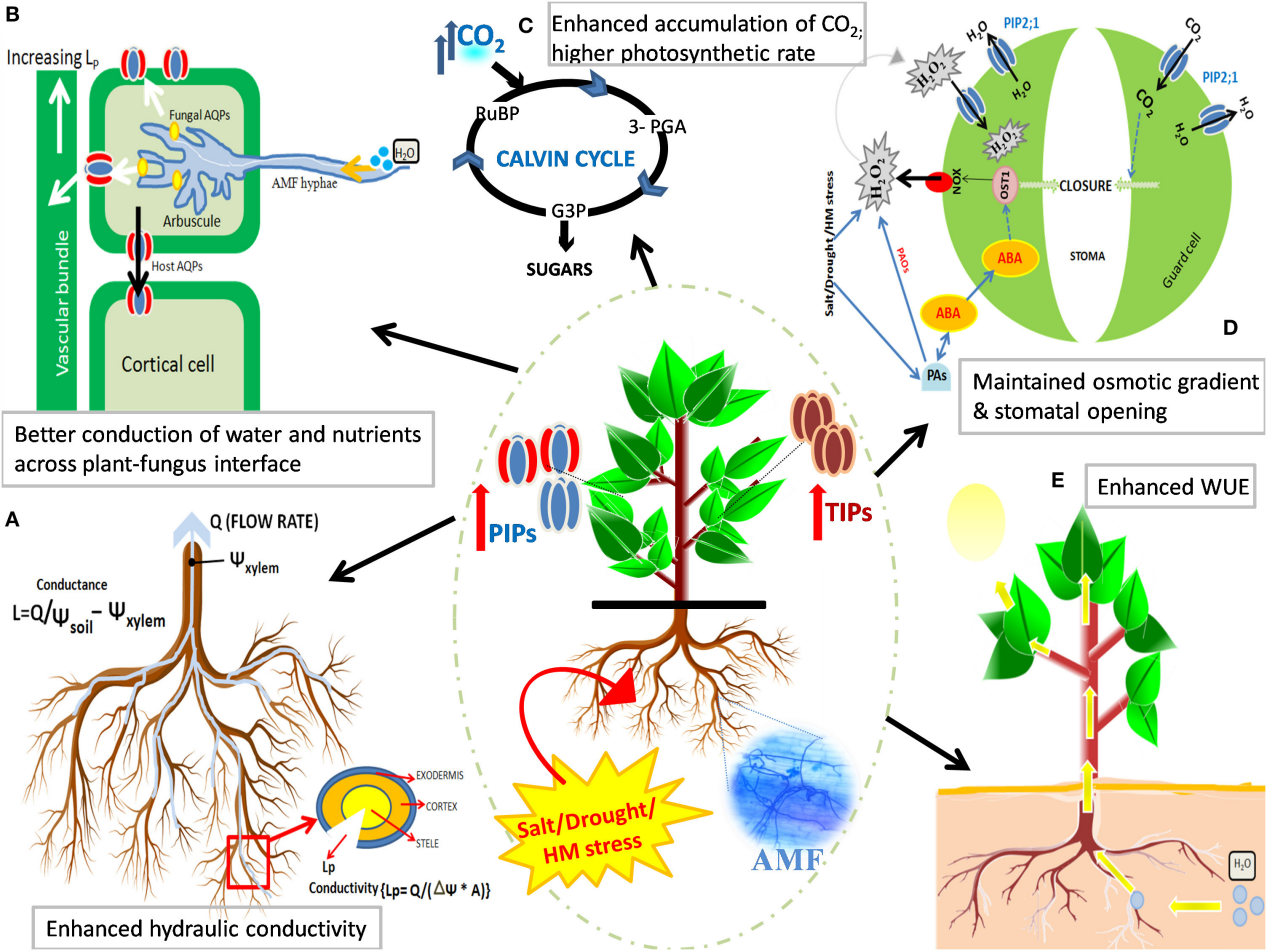
Effect of mycorrhizal symbiosis and abiotic stress on the regulation of aquaporin (AQP) expression and improving the stress tolerance in plants. AMF strengthen the regulation of *AQP* genes and modulates AQP protein to fortify tolerance of plants to water-deficit conditions arising from salt, drought, or metal stress. AMF-mediated upregulation of AQP activity [especially plasma membrane intrinsic proteins (PIPs) and tonoplast intrinsic proteins (TIPs)] results in: **(A)** an improved root hydraulic conductivity, **(B)** a better exchange of substrates and boosted nitrogen uptake across plant-fungal interface, **(C)** the accumulation of intracellular CO_2_, thus aiding in more photo-fixation of carbon (higher photosynthetic rates), **(D)** maintained osmotic gradient (and cell turgidity) and stomatal opening, and **(E)** improved transcellular conduction of water and plant water status by maintaining water-use efficiency (WUE).

Since AQPs are inherently regulated at transcriptional and posttranscriptional levels, it becomes important to delineate the exact role of AMF in the modulation of AQP-encoding genes as a mechanism to enhance the stress tolerance of plants under water-deficit conditions. However, the dynamics of AMF-mediated AQP regulation remain enigmatic to date. Abiotic stress, mycorrhization, or an interplay between the both evokes a signaling relay that begins with the generation of stress messengers and/or stress-induced hormones. We posit that AMF, under stress, might influence the AQP expression at transcriptional, translational, and post-transcriptional (phosphorylation, multimerization, cycling, and internalization of AQPs) levels that contributes to the overall increment in AQP expression and protein abundance, thus boosting the conduction of H_2_O, CO_2_, glycerol, NH_3_, etc., in stressed plants. AMF-reinforced regulation of AQP expression and transcript abundance can fortify tolerance of plants to water-deficit conditions arising from different stresses by improving root hydraulic conductivity, better exchange of nutrients across the plant–fungus interface, higher photosynthetic rates, maintaining cell turgidity and stomatal opening, and improving water-use efficiency of the plant (Figure 4).

As proposed by Javot and Maurel (2002), the ability of AMF to accelerate the water uptake in roots should imply enhanced permeability of water, which can be derived from higher activity of AQP genes; which is why mycorrhization should account for the upregulation of AQPs to promote transcellular water conduction. However, the results obtained in mycorrhized water-stressed soybean and lettuce plants were incongruous to the aforementioned hypothesis, as *PIP* expression was downregulated in both the plants colonized by mycorrhiza (*Glomus* spp.), although the leaf Ψ and RWC were higher in AM plants than their non-AM counterparts (Porcel et al., 2006). Such an effect of AM symbiosis might be conceived as a regulatory mechanism operative in AM plants to: (1) conserve metabolic energy under stressful conditions or (2) restrict water loss from the tissues into the hypertonic soil milieu. However, the downregulation of *PIP* genes was observed only in the case of plants inoculated with *G. mosseae* and not with *G. intraradices*. The results, somehow, correspond with the study of Marulanda et al. (2003), wherein the soil-water uptake capacity of *G. intraradices* was the highest and *G. mosseae* showed reduced efficiency thereof. This speaks for different strategies employed by the two fungi in order to safeguard plants from water stress. *G. intraradices* might be conditioned to enhance water permeability of roots and, thus, maintain higher expression of PIP aquaporins in the inoculated plants; while *G. mosseae* seems to be directing the conservation of already absorbed water in the plants, hence decreasing membrane water permeability by downregulating PIP gene expression (Ruiz-Lozano and Aroca, 2010).

In another study conducted by Porcel et al. (2005), the symbiotic efficiency (measured in terms of plant biomass), and not the root colonization efficiency, of the mycorrhized *NtAQP1* antisense tobacco plants was compromised on silencing the *NtAQP1* gene under drought stress. The results obtained have led to suggest the following: (1) *NtAQP1* is irrelevant in the colonization process, (2) the reduction in *NtAQP1* transcripts might have been compensated by other AQPs, and/or (3) *NtAQP1* is involved in CO_2_ diffusion in tobacco accounting to the promotion of photosynthesis and augmented production of photosynthates, resulting in better growth of plants (Uehlein et al., 2003; Bárzana et al., 2012). Symbiosis by *Rhizophagus irregularis* enhanced drought tolerance in *R. pseudoacacia* plants by calibrating the expression of *RpAQP* genes, and by improving plant biomass, cellular water status, net photosynthetic rate, and stomatal conductance (He et al., 2016). The expression levels of *RpTIP2;1* and *RpPIP2;1* were consistently augmented by AMF in all three tissues, leaves, stem, and roots during stress, thereby canalizing water fluxes toward plant tissues of significant importance in physiology. Differential expression of AQPs has also been reported in AMF-inoculated *Zea mays* (Bárzana et al., 2014) and *P. trifoliata* (Jia-Dong et al., 2019), manifested in altered physiology and plant growth performance under different regimes of drought stress (Table 2). Changes in the AQP gene expression profile under drought stress has been noticed not only in plants associated with AMF but also in the colonizing AM species thereof. The expression of two AQP genes, *GintAQPF1* and *GintAQPF2*, of *G. intraradices*, was significantly induced in response to water-deficit conditions, thus reinforcing the postulation of AMF involvement in directing plant tolerance to cellular desiccation by increasing water-use efficiency (WUE) (Li et al., 2013).

In maize colonized by *R. intraradices, ZmPIP2;2* and *ZmPIP2;6* were induced by the AMF along with the induced expression of fungal *GintAQPF2* under drought stress. AM inoculation resulted in an increased photosynthetic rate, an enhanced stomatal conductance, and a higher photosynthetic capacity. The phosphorylation levels of PIP2 were also found to be enhanced that might have translated into increased AQP activities in the mycorrhizal plants (Quiroga et al., 2019a). In mycorrhizal maize plants, the abundance of phosphorylated AQP proteins (PIP1, PIP2, PIP2A, PIP2B, and PIP2C) decreased in well-watered plants subjected to IAA or 6FI (inhibitor of IAA). However, not so significant changes were observed in the phosphorylation levels of AQPs in drought-stressed mycorrhizal plants (Quiroga et al., 2020) (Table 2). Similar observations were obtained in a previous study by Quiroga et al. (2019b), wherein no change in *ZmAQP* accumulation was observed in drought-stressed AM plants in the presence of sodium azide (metabolic inhibitor of AQPs). This could be attributed to the already higher apoplastic water flow and root hydraulic conductance in AM plants (over non-AM plants). Thus, the inhibitory effect of sodium azide on root hydraulic conductance in AM plants was lesser, which, together with increased apoplastic water flow, suggested a compensatory mechanism for the inhibition of AQP activity in these plants under stress (Quiroga et al., 2019b). Even so, Zou et al. (2019) observed that the expression of all *PtPIPs* in drought-stressed trifoliate orange was downregulated by the AMF treatment, while the *PtTIPs* expression remained unaltered under the same set of conditions. Increased overexpression of *PIPs* may accelerate wilting in plants; hence the decreased *PIP* expression in AMF-inoculated drought-stressed plants can be speculated as a mechanism to minimize water loss in stressed plants (Ruiz-Lozano and Aroca, 2017). Besides, a dramatic reduction in the root *PtNIP1;1; PtNIP5;1 and PtNIP6;1* levels were observed in water-stressed AM plants. As fungal hyphae can directly provide boron to the host plant, the decrease of NIPs is seen as a potential avoidance mechanism to the ensuing toxicity by otherwise excessive boron accumulation in the plant (Ruiz-Lozano and Aroca, 2017). In yet another study by Jia-Dong et al. (2019), both drought stress and AMF notably amplified the relative level of expression of root *PtTIP1;2, PtTIP1;3, and PtTIP4;1* in *P. trifoliata*, which might have accounted for an increased active water absorption. Contrastingly, the expression levels of *PtTIP2;1* and *PtTIP5;1* were found to be reduced in stressed AM plants. Since fungal hyphae can also meet the water needs of the host plant, this might be a strategy of AM plants to not rely on extensive host AQP expression and conserve energy. Moreover, increased ABA content observed in these plants could have hastened the water retention, thereby diminishing the requirement for overexpression of all the root-TIPs to absorb water (Table 2).

Taken together, it can be summarized that different AQP genes exhibit upregulation or downregulation under the same stress regime and those belonging to the same subgroup may register distinct expression profiles under different stress regimes (Bárzana et al., 2014). The expression pattern of same isoforms also differs with the fungal strain under study and the tissue analyzed. Despite vast experimental evidence about the implication of AQP genes in various stress responses, the explicit role of individual genes or specific AQP subfamilies is still baffling, owing to their extremely complex and integrated roles in response to various environmental stimuli, and active participation in fundamental growth and developmental processes. A comprehensive understanding of how AMF regulates AQP gene expression has pragmatic implications for crop improvement and management under stress.

### Heavy Metal Stress

As aforestated, the regulation of AQPs is governed by various factors including abiotic conditions like HMs. Metal ions influence the AQP activity by binding to the sulfhydryl groups of the proteins, inducing conformational changes (gating), and thus declining their water conduction capacity (Agre et al., 1998). Metal stress also affects the plant water status by impairing root growth, influencing stomatal density, decreasing vessel and tracheid size, all of which result in deceleration of short-distance and long-distance water conduction (Rucińska-Sobkowiak, 2016).

The regulatory influence of HMs on AQP activity and expression has been validated in several plants, like Hg-stressed *Pisum sativum* (Beaudette et al., 2007), Cu-stressed *Mesembryanthemum crystallinum* (Kholodova et al., 2011), arsenic [As (III)] exposed *Brassica juncea* (Srivastava et al., 2013), HMs (Zn^2+^, Pb^2+^, Cd^2+^, and Hg^2+^) stressed *Allium cepa* that accounted to metal-induced drop in water permeability in the order of Hg^2+^ > Cd^2+^ > Pb^2+^ > Zn^2+^ (Przedpelska-Wasowicz and Wierzbicka, 2011), Zn-stressed poplar (Ariani et al., 2019), and *Brassica rapa* (Fatemi et al., 2020). Multiple reports validate the significance of AQP activity as efflux transporters that excrete HMs out of the cellular space (Vats et al., 2021). For instance, *Oryza sativa NIP2;1* mediates As(III) efflux (Zhao et al., 2010), and NIPs from Arabidopsis participate in the expulsion of As (III) from the cell (Xu et al., 2015). Increased internalization and relocation of AQP proteins are also presumably associated with HM sequestration, and thus providing tolerance to the mechanism in plants (Vats et al., 2021). *NIP2;1* is a multifunctional AQP whose role in the transportation of As, B, and Si has been experimentally ratified (Yamaji and Ma, 2007; Ma et al., 2008; Schnurbusch et al., 2010). Grapevine *VvXIP1* is involved in the transportation of Cu, As, Ni, along with H_2_O_2_, thus might have a prospective role in metal homeostasis and signaling (Noronha et al., 2016).

Aquaporin response is the primary feedback to metal toxicity; and disturbed water balance is the first stress-triggered episode affecting the AQP confirmation (open/closed) (Przedpelska-Wasowicz and Wierzbicka, 2011). This offers the notion that AQPs have a key role to render under metal stress. Nevertheless, the clear mechanistic role furnished by different AQP isoforms under stress is yet to be categorically established owing to the complexity of integrated stress response that further varies with the plant species (Vats et al., 2021). Hence, the modulation of this recondite player could be a potential mechanism reinforced by AMF under HM stress, like that under salinity and drought. Unfortunately, the studies that corroborate this hypothesis and clarify the relation between plant–water relation and metal stress, and conceived role of AMF-mediated modulation of AQP activity in this context are slim to none. An exclusive study on *Salix purpurea* L. (a potential phytoremediator of trace elements) was conducted by Almeida-Rodríguez et al. (2016) wherein the symbiotic association of the host plant and *R. irregularis* was found to regulate a wide spectrum of metabolic and physiological outcomes upon Cu exposure. AMF-reinforced dynamic regulation of root AQPs, *PIP1;2*, together with an upregulation of root *TIP2;2* were held accountable for the maintained root hydraulic conductivity (L_P_) and leaf-specific conductivity (K_L_) under Cu stress. The observed translocation and accumulation of Cu ions in the cell wall and different organelles might be an outcome of osmoregulation encouraged by AQPs. TIPs and PIPs are key conduits of water conduction during this process (Table 2).

Although there has been profuse knowledge explosion in the domain of plant–AMF–HM interaction and multiple mechanisms have been ascribed to the ameliorative role of mycorrhizal association in assuaging HM stress, the arena of AMF-regulated AQP expression under metal stress is least explored. Hence, this might offer a potential area of research to decode the bona fide contribution of AMF to AQP regulation under metal stress. Moreover, the studies that deal with the functional characterization of fungal AQPs, which might serve as facultative AQPs to plants under stress, need to be performed. How fungal AQPs get affected under HM stress remains unexplored. Research flux should be trained toward studying the effect of metal toxicity on both host and fungal AQP genes and whether fungal AQPs are competent to counterbalance the metal-induced AQP downregulation in the host plants. More fungal AQP genes need to be identified and examined for their structure, function, and regulation under stress and otherwise.

## Conclusion and Perspective for Future Studies

Recurring episodes of environmental stresses affect arable land and stifle plant growth at both quantitative and qualitative levels. As opposed to biotic stress, which is controlled by monogenic trait, tolerance to abiotic stress is a multigenic complex trait that involves a multi-component signaling cascade, hence more difficult to monitor and engineer comparatively. Lately, several efforts are being invested in refining metal/salt/drought tolerance through the AMF application (mycoremediation) or genetic engineering. However, the complexity of tolerance mechanisms and difficulty in the transfer of technology to field conditions account to apparent inconsistency between theory and practice.

As reiterated in the paper, there exists a synergism between AMF and PA metabolism, which is exploited in plants under stressful conditions. However, the properties of signaling mechanisms, intricacies of cross-talk between PAs and other metabolic routes, and an exact mechanism underlying the putative role of AMF in mediating PA-induced alleviation of abiotic stress are far from being completely decoded owing to the increasing level of complexity. Considering that, the transcriptomic and metabolomic investigation in plants with knocked out/down PA synthesizing and catabolizing genes, interacting with AMF under stress, could offer a good outset for research direction. Besides, these approaches can be extrapolated to legumes wherein “rhizobia–AMF–PA” tripartite interaction can be investigated (Menéndez et al., 2019). The effect of PA knockout mutants can also be studied on other metabolic routes that are in a close-knit association with the PA metabolism under stress.

**Figure 5:**
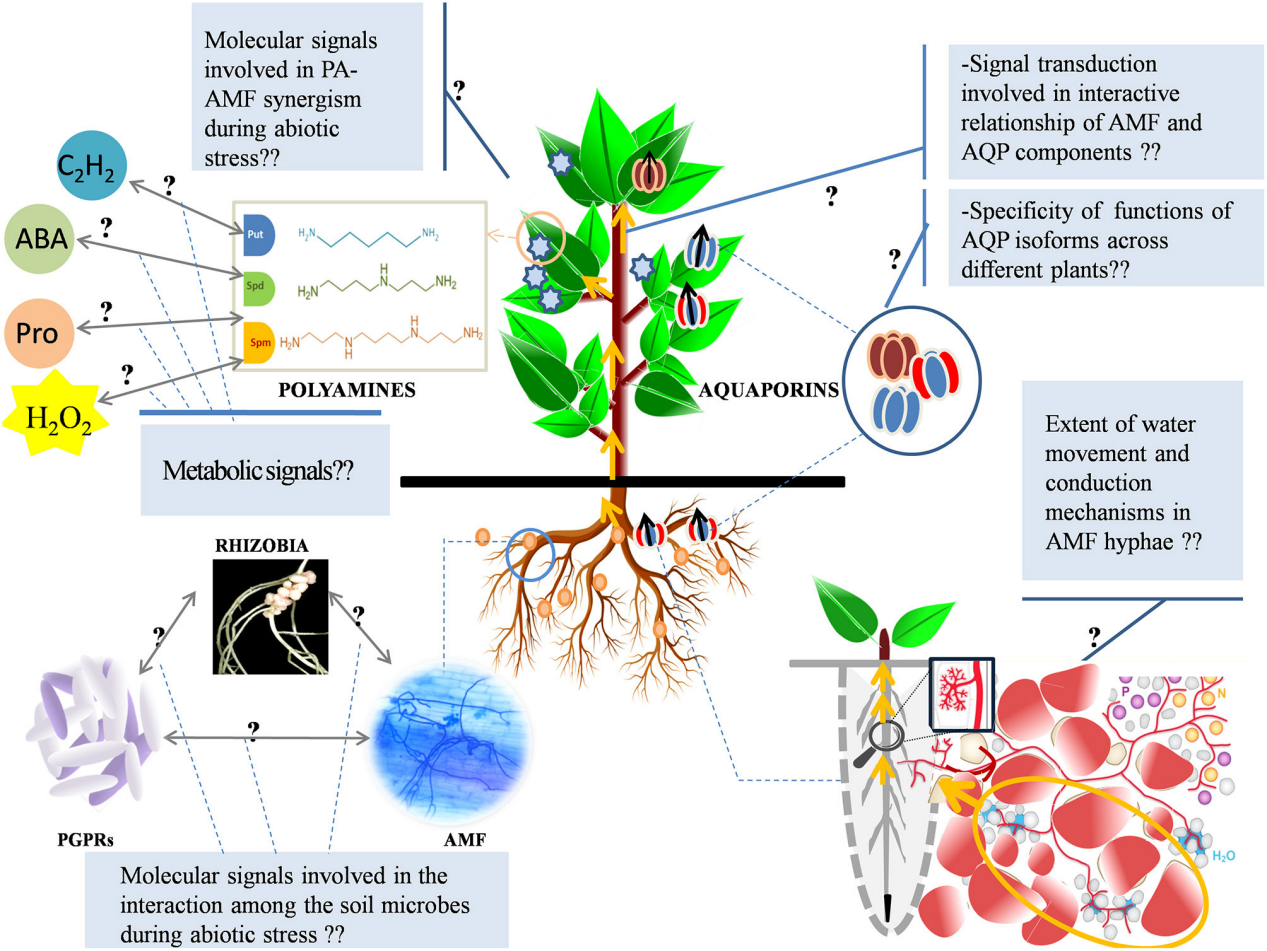
Probable knowledge gaps in the understanding of AMF and abiotic stress interaction on PAs and AQPs. The molecular signals involved in the interactivity of AMF with PAs and that of AMF with AQP components under stress are unexplored. The metabolic pathways that are involved in the cross-talk of PAs with other metabolic routes (ethylene, ABA, Pro, GABA, and H_2_O_2_) during stress need to be functionally dissected. Also, a deep understanding of the regulation of C flow into the N-metabolism pathways associated with salinity-induced modulation of PA levels is lacking and demands further exploration. The functional specificity of different AQP homologs across different species needs to be mapped (under stress or otherwise). Probable interaction (chemical signaling) among various beneficial soil microbes (AMF, PGPRs, and rhizobia) that facilitates plant tolerance/resistance to abiotic stresses is unknown and demands extensive exploration. Also, the specific function of AQP homologs in plant-microbe interactivity (positive or negative) is an area, which is relatively unplumbed. Fungal hyphae enhance water uptake from the mycorrhizosphere but the extent of translocation mediated by hyphae that eventually contributes to whole-plant water uptake (under salt stress or otherwise) is still abstruse. The dissection of water movement and conduction mechanisms in AM-fungal hyphae needs to be done, based on the direction and flux of water flow, with a special emphasis on the water drivers. ABA, Abscisic Acid; Pro, Proline; GABA, Gamma-Aminobutyric Acid; H_2_O_2_, Hydrogen Peroxide; AQP, Aquaporins; PA, Polyamine; AMF, Arbuscular Mycorrhizal Fungi; PGPRs, Plant Growth-Promoting Rhizobacteria.

The cellular and molecular mechanisms of mycorrhizal influence on the AQP expression profile (increase/decrease) in response to water-deficit conditions (imposed by salt, drought, and HM stress) remain elusive and need exhaustive research (Figure 5). These voids in understanding offer potential research landscapes in quest of deeper insights and practical implications of AMF in stress. In order to advance a step forward in understanding AQP-abiotic stress relations in AM plants, the expression of the same AQP members (isoforms) should be investigated across different AMF species and types of stress in order to discern the specificity in AQP function and nature of regulation by AMF. Furthermore, probing the effect of the same AMF species on the AQP regulation across a combination of biotic and abiotic stresses could be another potential research line. Such studies can be conducted on multiple “HM/salt/drought-tolerant” AMF species that are naturally found in saline or metalliferous environments in association with different crops, and then comparative analysis of their effects can be done in order to achieve the most efficacious combination of AMF species that can be employed as biofertilizers to dilute the ensuing toxic effects of stress on plants.

The fact that fungal hyphae enhance the water uptake from mycorrhizosphere to plant vasculature has been well-reviewed (Evelin et al., 2019), but the extent of translocation mediated by hyphae that eventually contributes to whole-plant water uptake (underwater deficit or otherwise) is still abstruse (Figure 5). On that account, the dissection of “water movement and conduction” mechanisms in AM-fungal hyphae needs to be done, based on the direction and flux of water flow with a special emphasis on the water drivers (AQPs, etc.) (Wu et al., 2013). Functional genomics can be integrated to expand the dimensions of our understanding on these facets. Identification of key mycorrhizal genes and TFs that contribute to the modulation of AQP activity should be identified, gauged, and engineered to improve crop health under stress and otherwise.

In plants, the stress-triggered damage can be attenuated by exploring and exploiting the potential of beneficial soil microbes [plant growth-promoting rhizobacteria (PGPRs), rhizobia, and AMF], and then employing them discretely or in combination. Numerous studies evince the positive influence of dual/triple inoculation on plant growth and tolerance under abiotic stress (Egamberdieva et al., 2013; Hashem et al., 2016; Hidri et al., 2019; Igiehon et al., 2020). Further research should focus on decoding the molecular signals that are involved in the interaction among the bio inoculants that ultimately influence plant physiology (Aroca and Ruiz-Lozano, 2009). The efficacy of multiple inoculations vs. single inoculation has also been investigated. Nonetheless, a lot more is there to understand about the fundamental mechanisms that are influenced by AMF and/or PGPR and/or rhizobia under stress (Yasmeen et al., 2019). A comprehensive molecular and physiological understanding of underlying mechanisms would enable us to better exploit the microbial bioresources as a potential tool for assuaging abiotic stress (Figure 5). Functional analysis of different growth-promoting microbes as first-generation plant biostimulants, when they are administered alone or in combination, would pave a way for the development of second-generation biostimulants with cooperative and complementary actions, mechanism of which can be functionally tailored. Besides, the employment of both microbial and non-microbial (seaweed extract or biochar) plant biostimulants should be promoted to reinforce sustainability efforts (González-González et al., 2020).

Global climate is changing rapidly than ever, menacing both plants and their symbiotic partners, thereby exerting significant direct and indirect effects on the growth and productivity of plants. Although AMF can attenuate the effects of abiotic stresses including climate change by fortifying the stress tolerance of host plants, their effects on AMF are given less attention, and hence poorly understood. Effects of these stresses on the symbiont could be direct or could be indirect through their impact on the host plant. It is expected that multiple abiotic stresses can directly affect the fitness, community composition, diversity, and symbiotic functioning of the fungi. Thus, keeping in mind the current scenario of global climate change and various other abiotic stresses, it is worth mentioning that studying the effects of abiotic stresses on AMF separately from plants will deliver a better understanding of the strengths and flaws of their ubiquitous relationship and for the employment of the AMF technology in sustainable agriculture.

In view of sustainable agriculture, the economical development of viable and potent AMF inocula to serve as bio fertilizer holds many practical applications; however, it is still inadequate due to limited research capacity and technical complexities. Primary obstacles to large-scale production of AMF inocula are the following: (1) the obligate nature of the fungi, which makes the phase of crop cultivation a prerequisite and (2) the fear of contamination by weeds and pathogenic spores in soil-based inocula. Conducting an expansive open-field inoculation treatment seems economically prohibitive. However, if AM-friendly management (fall cover cropping and conservation tillage) is executed and AM-biodiversity is well-established, the fungal community will perpetuate and the hyphal networks will remain unaltered (Lehman et al., 2012; Berruti et al., 2016). A major step toward stable usage of AMF is to conduct wholesale field trials at diverse locations and analyze the cost-economy (Ceballos et al., 2013) in order to better aware the potential users of the advantages of AMF. Additionally, autonomous production of customized inocula from the native soil *per se* can be encouraged so as to make the strategy of bio fertilization and stress amelioration more economical for farmers of developing countries.

Taken together, AMF must be scouted on all levels to further examine their beneficial roles in the natural environment where plants are subjected to multiple stresses at a time; this would be imperative to make complete use of AMF as a bio fertilizer and stress mitigator in order to achieve sustainable agriculture production and guarantee food security.

## Author Contributions

KS and RK conceptualized the design, draft, and layout of the literature work. All the authors have critically reviewed the manuscript and approved it for submission.

## Conflict of Interest

The authors declare that the research was conducted in the absence of any commercial or financial relationships that could be construed as a potential conflict of interest.

## Glossary

ABA, Abscisic Acid; ADC, Arginine Decarboxylase; As, Arsenic; Al, Aluminum; AM, Arbuscular Mycorrhiza; AMF, Arbuscular Mycorrhizal Fungi; APOX, Ascorbate Perroxidase; AQPs, Aquaporins; C, Carbon; Cad, Cadaverine; Cd, Cadmium; CHA, Cyclohexylamine; Ci, Intracellular CO_2_ Concentration; Cl^–^, Chloride Ion; cNMP, cyclic Nucleotide Monophosphate; Co, Cobalt; CO_2_, Carbon Dioxide; Cr, Chromium; Cu, Copper; CuAO, Copper Amine Oxidase; DAO, Diamine Oxidases; DFMA, α-Difluoromethyl Arginine; DFMO, α-Difluoromethyl Ornithine; DHAR, Dehydroascorbate Reductase; DNA, Deoxyribonucleic Acid; ER, Endoplasmic Reticulum; Fe, Iron; GA, Gibberellic Acid; GABA, Gamma-Aminobutyric Acid; GABAT, Gamma-Aminobutyric Acid Transaminase; Gs, Stomatal Conductance; GSH, Glutathione Reduced; GSSH, Glutathione Oxidized; H_2_O, Water; H_2_O_2_, Hydrogen Peroxide; Hg, Mercury; HMs, Heavy Metals; *HMT*, Heavy Metal Tolerance Gene; J_v_, Sap Flow Rate; KIRC, Inward Rectifying K^+^ Channels; K_L_, Leaf-Specific Conductivity; L_h_, Hydrostatic Root Hydraulic Conductance; L_o_, Root Hydraulic Conductance; L_t_, Leaf Temperature; MDHAR, Monodehydroascorbate Reductase; MIPs, Major Intrinsic Proteins; Mn, Manganese; Mo, Molybdenum; MT, Metallothionien; MVBs, Multivesicular Bodies; N, Nitrogen; Na^+^, Sodium Ion; NaCl, Sodium Chloride; *NCED: 9-cis-epoxycarotenoid dioxygenase*); NH_2_, Amine Group; NH_3_, Ammonia; Ni, Nickel; NIPs, Nodulin-26 like Intrinsic Proteins; NO, Nitric Oxide; ODC, Ornithine Decarboxylase; PAO, Polyamine Oxidase; PAs, Polyamines; Pb, Lead; PCs, Phytochelatins; PGPRs, Plant Growth-Promoting Rhizobacteria; PIPs, Plasma Membrane Intrinsic Proteins; POP, Persistent Organic Pollutants; Put, Putrescine; RNA, Ribonucleic Acid; RWC, Relative Water Content; SA, Salicylic Acid; SAM, S-Adenosyl Methionine; SAMDC, SAM Decarboxylase; SAR, Sodium Adsorption Ratio; SIPs, Small Basic Intrinsic Protein; Si, Silicon; Spd, Spermidine; SPDS, Spermidine Synthase; Spm, Spermine; SPMS, Spermine Synthase; TCA, Tricarboxylic Acid Cycle; TGN, Trans-Golgi Network; TIPs, Tonoplast Intrinsic Proteins; WUE, Water-Use Efficiency; Zn, Zinc; Ψ, Water Potential.
